# FGF7 Polymorphisms, Especially Chr7:57803771:C>T, are Associated with Lambskin Traits in Hu Sheep

**DOI:** 10.3390/ani16193126

**Published:** 2026-10-05

**Authors:** Kebede Habtegiorgis Beshah, Jing Xie, Daxiang Wang, Bingru Zhao, Muhammad Tariq, Abdul Quddus, Dagan Mao

**Affiliations:** 1 College of Animal Science and Technology, Nanjing Agricultural University, Nanjing 210095, China; 2024205061@stu.njau.edu.cn (K.H.B.); 2023105023@stu.njau.edu.cn (J.X.); zhaobingru@njau.edu.cn (B.Z.); tariq@stu.njau.edu.cn (M.T.); 2024205060@stu.njau.edu.cn (A.Q.); 2 Debre Birhan Agricultural Research Center, Amhara Regional Agricultural Research Institute, Debre Birhan P.O. Box 112, Ethiopia; 3 Jiangsu Qianbao Animal Husbandry Co. Ltd., Yancheng 224000, China; wdx911012@163.com

**Keywords:** Hu sheep, lambskin traits, *FGF7*, SNP, marker-assisted selection

## Abstract

Lambskin quality is an economically important trait in Hu sheep (*Ovis aries*), with wool-pattern characteristics contributing substantially to commercial value. However, the genetic basis of lambskin variation remains incompletely understood. This study evaluated single nucleotide polymorphisms (SNPs) in four candidate genes involved in hair follicle development, *FGF7*, *FGFR2*, *SMAD5*, and *SOSTDC1*, in 384 Hu sheep using Sequenom MassARRAY^®^ technology. Allele and genotype frequencies, heterozygosity, polymorphism information content (*PIC*), and Hardy–Weinberg equilibrium (HWE) were assessed, followed by association analyses with categorical and quantitative lambskin traits. A total of 18 polymorphic SNPs were successfully genotyped. Three *FGF7* SNPs showed significant associations with lambskin traits, with all corresponding significant allelic associations remaining significant after false discovery rate (FDR) correction. For Oar_v4.0:Chr7:57803771:C>T, the C-allele frequency increased from 0.30 in straight-wool individuals to 0.53 in fully wavy-wool individuals, and the C allele was associated with higher odds of the fully wavy phenotype (OR = 2.70, 95% CI: 1.56–4.68). This locus was also significantly associated with pattern area and lambskin grade, whereas Oar_v4.0:Chr7:57740946:G>A and Oar_v4.0:Chr7:57741376:T>G were associated with pattern area and pattern width. *SMAD5* variants showed nominal associations with some traits but did not remain significant after FDR correction, whereas *FGFR2* showed no significant associations and *SOSTDC1* showed a nominal genotype association with lambskin grade. Protein–protein interaction analysis further placed *FGF7* within a network involving signaling proteins related to epithelial proliferation and hair follicle development. Collectively, these findings identify the *FGF7* region, particularly Oar_v4.0:Chr7:57803771:C>T, as the strongest candidate signal associated with lambskin variation in Hu sheep.

## Simple Summary

Hu sheep is a Chinese breed which is well known for its distinctive white lambskin and attractive wavy pattern type. Lambskin is the wool-bearing pelt of young lambs. Pattern type, pattern width, and pattern area of the wave are important characteristics used to evaluate lambskin quality and are closely related to hair follicle development. However, the genetic factors contributing to differences in these traits are not yet fully understood. In this study, we investigated four genes that may be involved in wool and hair follicle development in 384 Hu sheep lambs and examined whether genetic differences in these genes were related to lambskin characteristics. Several genetic differences were associated with lambskin traits, with the clearest associations observed in the *FGF7* gene region. We further examined how *FGF7* proteins may interact with other proteins involved in related biological processes. In particular, the *FGF7* variant Oar_v4.0:Chr7:57803771:C>T was associated with pattern area and lambskin grade. These findings provide additional information about the genetic basis of lambskin variation in Hu sheep and provide a foundation for future studies of the molecular mechanisms underlying lambskin pattern formation.

## 1. Introduction

Sheep (*Ovis aries*) are a major source of various agricultural products, including meat, wool, skin, and milk [1]. Lambskin, the hide obtained from young sheep, is a high-value natural material due to its fine grain, softness, elasticity, and light weight [2,3]. Owing to these superior physical properties, lambskin holds considerable importance in both livestock systems and the global leather industry. Recent evidence indicates sustained growth in global lambskin production, reflecting increasing consumption of leather goods and expansion of high-value markets [4,5]. Moreover, lambskin is estimated to account for a notable share of the leather goods sector, underscoring its continued relevance in modern market trends. In parallel, growing emphasis on sustainability, traceability, and ethical sourcing is reshaping the industry, positioning lambskin as a key material within emerging frameworks of responsible and high-quality leather production [4].

Over the past three decades, China has become the world’s largest sheep producer. Hu sheep are currently recognized as one of the prioritized breeds by the Ministry of Agriculture and Rural Affairs of China [4,6]. This breed is particularly valued for its high-quality lambskin, which is characterized by distinct wave patterns, fine fiber texture, and a desirable curl structure [7]. Hu sheep also have important production value, and recent research has examined nutritional strategies to improve their growth performance, carcass traits, and meat quality [8]. As these traits are quantitatively inherited and can be affected by developmental and environmental factors, improvement through conventional phenotypic selection alone remains relatively slow.

Lambskin pattern type and formation are closely related to hair follicle morphogenesis. Hair follicles are dynamic mini-organs that cycle through anagen, catagen, and telogen phases; variation in follicle growth, differentiation, and curvature directly underpins diversity in hair length, texture, and wave pattern [2,9,10]. In Hu sheep, transcriptome-based studies have shown that differences in lambskin pattern are associated with broad changes in hair follicle gene expression, including genes involved in epithelial differentiation, follicle development, and extracellular signaling [11]. Previously microarray analysis identified differential expression signatures among follicles producing large, medium, and small wave patterns, and subsequent integrated mRNA-miRNA and lncRNA analyses further supported the view that lambskin patterning is underpinned by a robust molecular and regulatory architecture [12,13].

Several classical signaling pathways regulate hair follicle development and skin appendage morphogenesis, including Wnt/β-catenin, TGF-β/BMP, Sonic Hedgehog, and FGF signaling [9,14,15]. Recent reviews and mechanistic studies indicate that *FGF7* [16,17] and *FGFR2* [18] promote keratinocyte proliferation, support follicular epithelial activity, and contribute to telogen-to-anagen transition and follicular regeneration processes that may affect lambskin-related traits [19,20]. In parallel, members of the *SMAD* family mediate intracellular signaling downstream of the TGF-β superfamily, including BMP pathways that are crucial for epidermal differentiation and hair follicle development. Specifically, *SMAD5* is involved in BMP/TGF-β signaling pathways to regulate cellular differentiation [9,15,21]), while *SOSTDC1* functions as a modulator of both BMP and Wnt signaling [22,23].

Candidate gene association analysis based on single nucleotide polymorphisms (SNPs) has been widely applied in livestock to identify markers for economically important traits, including reproduction, wool quality, and growth [19,24,25]. Therefore, to identify informative molecular markers and elucidate the genetic architecture underlying lambskin quality traits in Hu sheep, the present study evaluated SNPs in four biologically plausible candidate genes, namely *FGF7*, *FGFR2*, *SMAD5*, and *SOSTDC1,* and their associations with lambskin quality traits in a Hu sheep population. The phenotypes examined included both ordered categorical outcomes, with pattern area scored from 0/4 to 4/4, representing 0% to 100% wavy area, and continuous measurements, including pattern width and sacral wool length. For each SNP, we determined genotype and allele frequencies, estimated genetic diversity parameters including observed and expected heterozygosity and polymorphism information content (*PIC*), and assessed conformity to Hardy–Weinberg equilibrium. Association analyses between SNPs and lambskin quality traits were performed using appropriate statistical models with adjustment for relevant covariates, including season, sex, and sibling structure. Additionally, protein–protein interaction (PPI) network analysis was conducted to further characterize the functional relationships of the candidate genes and to place the identified associations within the broader molecular pathways involved in hair follicle development and lambskin formation. The results will facilitate molecular breeding and contribute to the sustainability of sheep farming.

## 2. Materials and Methods

### 2.1. Ethical Statement

All procedures involving animals were approved by the Institutional Animal Care and Use Committee of Nanjing Agricultural University (Permit No. SYXK (SU) 2022-0031; approved on 1 December 2022) and were conducted in accordance with the Regulations for the Administration of Affairs Concerning Experimental Animals of the People’s Republic of China.

### 2.2. Animals and Phenotypic Data

Phenotypic data and tissue samples were collected from 384 Hu lambs at Jiangsu Qianbao Animal Husbandry Co., Ltd. (Yancheng, China). All animals were raised under standardized management and feeding regimens on the same farm to minimize environmental variation. Lambskin traits were evaluated within 3 days of age in accordance with the national standards GB/T 4631-2006 [26] for Hu Sheep and GB/T 14629.1-2018 [27] for Hu sheep lambskin. The evaluated traits comprised pattern type, pattern width, pattern area, and sacral wool length. Lambskin grade was assigned according to the relevant national criteria for Hu sheep lambskin. Grade 1 represents the highest grade, characterized by a fully developed wavy pattern and short sacral wool, whereas higher numerical grades indicate progressively lower grades. Pattern area was scored as the proportion of the lambskin surface exhibiting a distinct, continuous wave pattern, on a scale from 0/4 (fully straight) to 4/4 (fully wavy). Sacral wool length was measured at the mid-dorsal region using a precision caliper (±1 mm). Additionally, fixed effects including sex, litter size (single, twin, triplet, or quadruplet), and season were recorded for each animal.

### 2.3. DNA Extraction

Genomic DNA was extracted from ear tissue samples using the standard phenol–chloroform method as described [28]. DNA concentration and purity were evaluated using a NanoDrop 2000 spectrophotometer (Thermo Fisher Scientific, Waltham, MA, USA). Samples with a DNA concentration >50 ng/μL and an A260/A280 ratio of 1.8–2.0 were considered qualified and stored at −20 °C. Qualified DNA samples were subsequently normalized to a uniform working concentration for MassARRAY^®^ SNP genotyping (Agena Bioscience, San Diego, CA, USA) [25,29,30].

### 2.4. SNP Selection

Candidate genes associated with hair follicle development and lambskin traits were selected based on previous studies and findings from whole-genome resequencing data. Within these candidate genes,18 SNP loci located in *SOSTDC1*, *SMAD5*, *FGF7*, and *FGFR2* were selected for genotyping (Table 1). The selection focused on polymorphic loci identified within the candidate genes from the available genetic information and whole-genome resequencing data. SNP annotations, including chromosome position, allele variation, and flanking sequences, were retrieved from the Oar_v4.0 reference genome (GCF_000298735.2) available in the National Center for Biotechnology Information (NCBI) database (https://www.ncbi.nlm.nih.gov/assembly/GCF_000298735.2/; accessed on 2 July 2026). Each SNP was classified according to its genomic context, including the 5′ untranslated region (5′ UTR) and 3′ untranslated region (3′ UTR). Flanking regions of 100 bp upstream and downstream of each target locus were extracted to provide sufficient local sequence context for MassARRAY assay design and primer selection (Agena Bioscience, San Diego, CA, USA). The 100-bp flanking sequence was used solely for assay design and was not a criterion for SNP selection. Detailed information for all SNP loci, including full Oar_v4.0 identifiers, chromosome numbers, physical positions, allele variations, and genomic contexts, is provided in Supplementary Table S1.

### 2.5. Genotyping

According to the reference sequences of ovine *SOSTDC1*, *SMAD5*, *FGF7*, and *FGFR2* genes in the Oar_v4.0 sheep assembly (GenBank assembly accession: GCF_000298735.2), PCR amplification primers and single-base extension primers for 18 candidate SNP loci were designed using MassARRAY Assay Design software v3.1 (Agena Bioscience, San Diego, CA, USA). All primers were synthesized commercially (Beijing, China). Genotyping was performed on the MassARRAY^®^ platform (Agena Bioscience, San Diego, CA, USA; formerly Sequenom) based on matrix-assisted laser desorption/ionization time-of-flight mass spectrometry (MALDI-TOF MS), following the manufacturer’s standard protocol [31].

Briefly, multiplex PCR amplification was conducted using locus-specific primers, followed by shrimp alkaline phosphatase (SAP) treatment to remove unincorporated dNTPs. Single-base extension reactions were subsequently carried out using mass-modified extension primers, and the final products were analyzed using the MassARRAY Analyzer (Agena Bioscience, San Diego, CA, USA). Genotype calling and quality control were performed using Typer software v4.0 (Agena Bioscience, San Diego, CA, USA). All 18 polymorphic SNPs across the four candidate genes were retained for downstream population genetic and association analyses. Primer sequences for the 18 SNP loci are provided in Supplementary Table S2.

### 2.6. Population Genetic Analysis

For each SNP, genotype and allele frequencies were calculated. Observed heterozygosity (*H_o_*), expected heterozygosity (*H_e_*), effective allele number (*N_e_*), and *PIC* were computed using standard formulas:

Allele frequencies:

*p* and *q* are allele frequencies (for a bi-allelic SNP, *p* + *q* = 1).

Observed heterozygosity:

$$H_{o}=\frac{N_{\mathrm{heterozygotes}}}{N}$$

$H_{e}=1-\sum p_{i}^{2}$ (for bi-allelic SNP: *H_e_* = 1 − (*p*^2^ + *q*^2^) = 2*pq*).

Effective allele number:

$$N_{e}=\frac{1}{\sum p_{i}^{2}}\;(\mathrm{for}\;\mathrm{bi}\text{-}\mathrm{allelic}\;\mathrm{SNP}:\;N_{e}=\frac{1}{p^{2}+q^{2}})$$

$$\mathrm{PIC}=1-\sum p_{i}^{2}-\sum \sum 2p_{i}^{2}p_{j}^{2}\;\mathrm{or}\;\mathrm{PIC}=1-(p^{2}+q^{2})-2p^{2}q^{2}$$

HWE was tested for each SNP using a chi-square test, and SNPs deviating strongly from HWE (*p* < 0.05) were flagged for interpretation depending on population structure and selection [32]. Allele and genotypic frequencies were compared across the five lambskin-grade categories and across the five pattern-area categories [33].

### 2.7. Association Analysis Between SNPs and Lambskin Traits

Association analyses were performed using a general linear model (GLM) implemented in SPSS 20.0. The statistical model was defined as:

*Y_ijkl_* = *μ* + *G_i_* + *S_j_* + *L_k_* + *M_l_* + *e_ijkl_*

where *Y_ijkl_* is the observed phenotypic value, *μ* is the overall mean, *G_i_* is the fixed effect of genotype, *S_j_* is the fixed effect of sex, *L_k_* is the fixed effect of siblings, *M_l_* is the fixed effect of birth month, and *e_ijkl_* is the random residual error. When genotype effects reached significance, pairwise comparisons were performed using Tukey’s honestly significant difference (HSD) test. To account for multiple comparisons across SNPs, false discovery rate (FDR) correction was applied using the Benjamini–Hochberg procedure. Statistical significance was declared at an FDR-adjusted *p*-value < 0.05.

### 2.8. Protein–Protein Interaction Network Analysis

For SNP loci significantly associated with lambskin traits, the corresponding candidate gene *FGF7* was subjected to protein–protein interaction (PPI) network analysis [25]. The PPI network of *FGF7* was constructed using the STRING database v12.0 (https://string-db.org), which integrates experimentally validated and computationally predicted protein interactions derived from experimental data, curated databases, co-expression analysis, gene neighborhood, gene fusion, and text mining. The *Ovis aries* species filter was selected, and the default confidence score threshold was applied. Only interactions supported by experimental evidence, curated databases, or high-confidence prediction algorithms were retained for interpretation. The resulting interaction network was analyzed to explore the potential biological functions and signaling pathways associated with *FGF7*, particularly those implicated in hair follicle development, epidermal proliferation, and skin appendage biology.

## 3. Results

### 3.1. Genetic Diversity and Polymorphism Information

**Figure 1:**
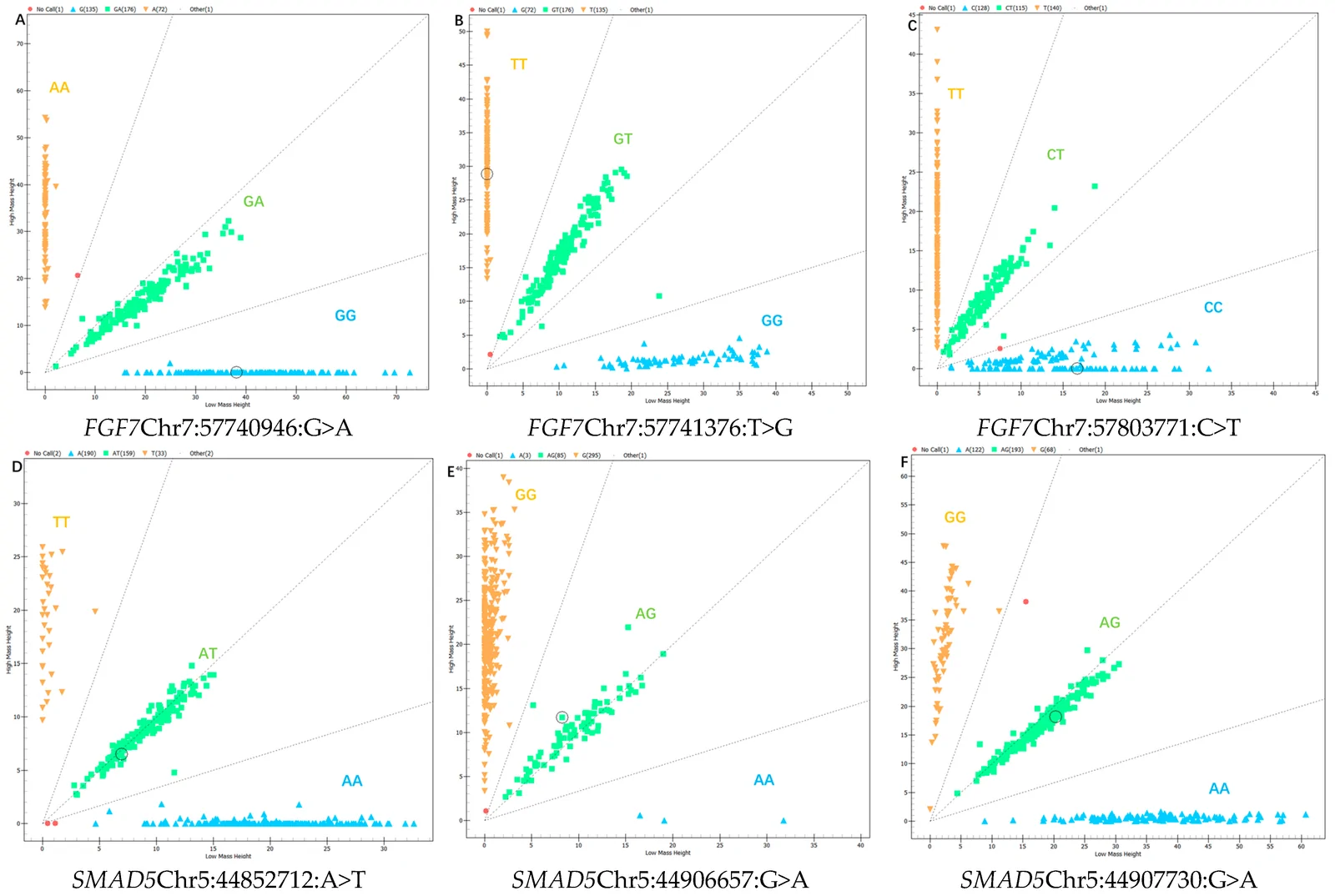
Representative MassARRAY^®^ SNP genotyping clustering plots for six SNP loci in Hu sheep. (**A**) *FGF7* Chr7:57740946:G>A; (**B**) *FGF7* Chr7:57741376:T>G; (**C**) *FGF7* Chr7:57803771:C>T; (**D**) *SMAD5* Chr5:44852712:A>T; (**E**) *SMAD5* Chr5:44906657:G>A; (**F**) *SMAD5* Chr5:44907730:G>A. In each panel, the *x*-axis represents the mass height of the extended primer product, and the *y*-axis represents the mass height of the internal control. Different colors indicate distinct genotype calls: orange (homozygous for allele 1), green (heterozygous), and blue (homozygous for allele 2).

A total of 18 polymorphic SNPs across the four candidate genes were successfully genotyped in 384 Hu sheep. Representative MassARRAY^®^ clustering plots for six SNP loci are shown in Figure 1, with clear separation of the three genotype clusters (two homozygous and one heterozygous genotype classes). Genetic diversity parameters for all polymorphic SNPs are summarized in Table 2. Minor allele frequencies (MAFs) ranged from 0.0794 (Chr5:44908255:A>G in *SMAD5*) to 0.4948 (Chr22:40347084:G>A in *FGFR2*). Based on MAF, 11 SNPs (61.1%) showed moderate polymorphism (MAF > 0.25), while 7 SNPs (38.9%) exhibited low polymorphism (MAF < 0.25). Observed heterozygosity (*H_o_*) ranged from 0.1276 (Chr5:44908255:A>G) to 0.5300 (Chr22:40346469:G>C), while expected heterozygosity (*H_e_*) ranged from 0.1462 to 0.4999. The effective number of alleles (*N_e_*) varied from 1.1713 to 1.9998. The polymorphism information content (*PIC*) values classified the SNPs into two categories: moderate informativeness (0.25 ≤ PIC ≤ 0.5) for 11 SNPs (61.1%), and low informativeness (*PIC* < 0.25) for 7 SNPs (38.9%). Hardy–Weinberg equilibrium (HWE) testing revealed that most loci conformed to equilibrium (*P* > 0.05); the exceptions were Chr7:57803771:C>T (*FGF7*) and Chr5:44908255:A>G (*SMAD5*), which deviated significantly from expected genotype frequencies (*P* < 0.05; Table 2).

### 3.2. Allele Frequency Distribution

The allele frequencies of the variants showing significant or suggestive allelic associations across pattern area and lambskin grade categories are presented in Table 3. In addition, the corresponding association statistics, odds ratios, confidence intervals, and FDR-adjusted *q*-values were summarized in Table 4.

Across pattern-area categories, *FGF7* Chr7:57803771:C>T showed an increase in C allele frequency from 0.30 in the 0/4 (straight) group to 0.53 in the 4/4 (fully wavy) group (χ^2^ = 19.30, *P* = 0.001). The same allele rose across lambskin grades from 0.30 in Grade 1 to a peak of 0.53 in Grade 3 (χ^2^ = 15.70, *P* = 0.003) (Table 4). The C allele was associated with increased odds of the fully wavy pattern (OR = 2.70, 95% CI 1.59–4.62) and of a higher lambskin-grade category (OR = 2.62, 95% CI 1.48–4.65). Both associations remained significant after FDR correction.

For *FGF7* Chr7:57740946:G>A, the G allele increased from 0.46 in the 0/4 group to 0.65 in the 4/4 group (χ^2^ = 18.74, *P* = 0.001), with OR = 0.47 (95% CI 0.28–0.77), remaining significant after correction (*q* = 0.011). The same pattern was observed for *FGF7* Chr7:57741376:T>G (χ^2^ = 18.75, *P* = 0.001; OR = 0.47, 95% CI 0.28–0.77; *q* = 0.011). Note that the OR below 1 for these two loci indicates that the allele increasing toward the wavy group is associated with lower odds of the reference (straight) pattern. Because the two variants displayed identical allele-frequency trajectories and closely corresponding genotype distributions, these associations may reflect strong linkage disequilibrium within the *FGF7* region; they should be interpreted as an *FGF7*-regional signal rather than two independent associations. Across lambskin grades, the G allele for Chr7:57740946:G>A was 0.46, 0.50, 0.64, 0.59 and 0.61 from Grades 1 to 5, and the corresponding values for Chr7:57741376:T>G were identical (χ^2^ = 10.00, *P* = 0.040 and χ^2^ = 10.07, *P* = 0.039, respectively). These two grade associations were nominally significant but did not remain significant after FDR correction.

For *SMAD5* Chr5:44852712:A>T, the A allele ranged from 0.65 in Grade 1 to 0.74 in Grade 4 (χ^2^ = 12.02, *P* = 0.017; OR = 1.11, 95% CI 0.63–1.95), an association that did not survive FDR correction (*q* = 0.124). *SMAD5* Chr5:44906657:G>A showed a nominal grade association (χ^2^ = 9.62, *P* = 0.047; OR = 0.62, 95% CI 0.31–1.24), which also did not survive correction (*q* = 0.213). Neither *SMAD5* variant showed a significant pattern-area association (*P* = 0.086 and *P* = 0.101, respectively).

In total, the allele-frequency analyses yielded eight nominally significant SNP–trait associations (*P* < 0.05): six involving *FGF7* (three pattern-area, one grade at *q* = 0.031, and two additional grade associations at *P* = 0.039–0.040 that were not FDR-significant) and two *SMAD5* grade associations. After FDR correction, only the three *FGF7* pattern-area associations in Table 4 (*q* = 0.011) and the *FGF7* Chr7:57803771:C>T grade association (*q* = 0.031) remained significant. Allele frequencies of all polymorphic SNPs across pattern area and grade categories are provided at Supplementary Table S3.

### 3.3. Genotype Frequency Distribution

Genotype frequencies of the three *FGF7* SNPs and one *SOSTDC1* SNP across the five pattern-area groups (0/4–4/4) and five lambskin-grade categories (Grades 1–5) are presented in Table 5. For *FGF7* Chr7:57741376:T>G, the frequency of the GG genotype decreased progressively from 0.32 in the 0/4 group to 0.14 in the 4/4 group, whereas the TT genotype increased from 0.24 to 0.43 toward the fully wavy group. The genotype distribution differed significantly across pattern-area categories (χ^2^ = 20.449, *P* = 0.0086) but not across lambskin grades (χ^2^ = 14.105, *P* = 0.0791). For *FGF7* Chr7:57740946:G>A, the AA genotype decreased from 0.32 in the 0/4 group to 0.14 in the 4/4 group, whereas the GG genotype increased from 0.24 to 0.43 toward the fully wavy group. The association with pattern area was significant (χ^2^ = 20.226, *P* = 0.0097), whereas the association with lambskin grade was not significantly (χ^2^ = 14.048, *P* = 0.0808). For *FGF7* Chr7:57803771:C>T, the CC genotype increased from 0.11 in the 0/4 group to 0.36 in the 4/4 group, whereas the TT genotype decreased from 0.51 to 0.29; the association with pattern area was significant (χ^2^ = 25.468, *P* = 0.0012). Across lambskin grade, TT genotype decreased from 0.51 in Grade 1 to 0.29 in Grade 5; the association with lambskin grade was significant (χ^2^ = 16.169, *P* = 0.0401).

For *SOSTDC1* Chr4:25022929:A>T, the AA genotype was the most frequent genotype across all pattern-area groups, with frequencies ranging from 0.50 to 0.56, whereas the TT genotype occurred at lower frequencies, ranging from 0.03 to 0.19. The association with pattern area was not significant (χ^2^ = 14.167, *P* = 0.0778). Across lambskin grades, the AA genotype was more frequent in Grade 4 (61%) and Grade 5 (53%), whereas the TT genotype remained uncommon (6.0–19.0%); the genotype distribution differed significantly across grade categories (χ^2^ = 16.111, *P* = 0.0410). The *SOSTDC1* genotype–lambskin grade association (*P* = 0.0410) was significant. Complete genotypic frequency for all polymorphic SNPs is provided at Supplementary Table S4.

### 3.4. Association Analysis of SNPs with Lambskin Traits

Associations between SNP genotypes and two lambskin traits, sacral wool length and pattern width, are presented in Table 6. Using the general linear model described in Section 2.7, three *FGF7* variants were associated with pattern width. For Chr7:57740946:G>A, the AA genotype was associated with a narrower pattern width than the AG and GG genotypes (overall *P* = 0.0021; Tukey’s HSD, *P* < 0.01). Similarly, for Chr7:57741376:T>G, the GG genotype showed a narrower pattern width than the GT and TT genotypes (overall *P* = 0.0022; Tukey’s HSD, *P* < 0.01).

Chr7:57803771:C>T, the CC genotype had a wider pattern than the TT genotype (overall *P* = 0.0039; Tukey’s HSD, *P* < 0.01). The CT genotype did not differ significantly from either homozygous genotype. The two variants Chr7:57740946:G>A and Chr7:57741376:T>G displayed identical genotype-group sample sizes and highly similar genotype-specific means; therefore, their associations may reflect strong linkage disequilibrium and should not be interpreted as independent signals without further analysis.

A nominal association was also observed between Chr5:44907730:G>A in *SMAD5* and sacral wool length (*P* = 0.0152), with the GG genotype showing a higher mean value than the AA and AG genotypes. Chr5:44906657:G>A in *SMAD5* showed a nominal overall association with pattern width (*P* = 0.0229; Table 6), but no Tukey-adjusted pairwise comparison was significant. Moreover, the AA genotype was represented by only three animals, limiting the reliability of this result. The remaining 13 SNPs showed no nominal association with either pattern width or sacral wool length. Detailed information of association between SNP genotypes and quantitative lambskin traits in Hu sheep is provided in Supplementary Table S5.

### 3.5. Protein–Protein Interaction Network Analysis of FGF7

**Figure 2:**
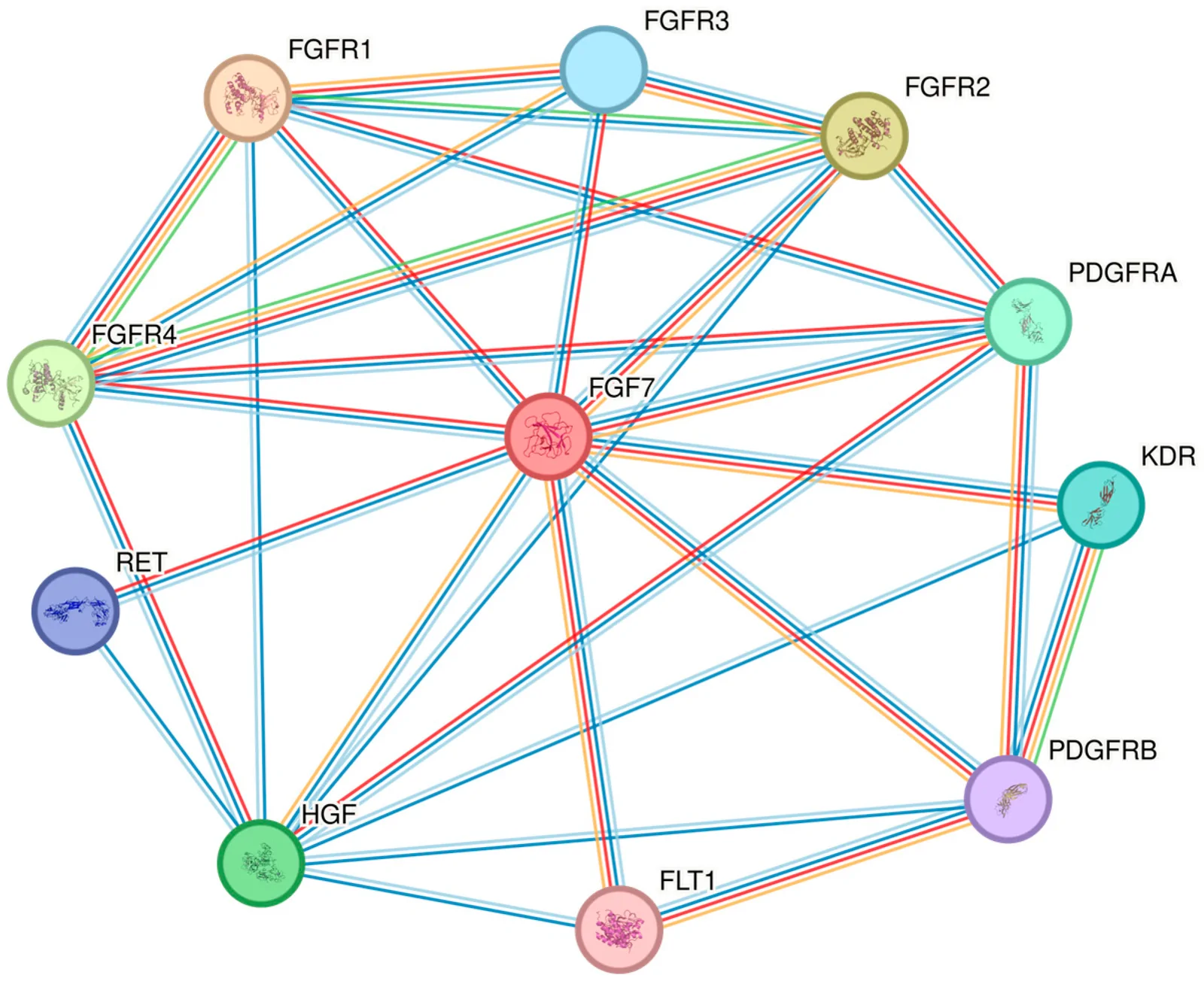
Protein–protein interaction network of *FGF7* predicted using the STRING database (v12.0) for *Ovis aries*. Nodes represent proteins and edges represent functional associations derived from experimental evidence, curated databases, and co-expression data.

To further characterize the interaction partners of *FGF7*, a protein–protein interaction (PPI) network was constructed using the STRING database (version 12.0) for *Ovis aries*. The resulting network is presented in Figure 2, and the 10 proteins with the highest interaction scores are summarized in Table 7. *FGF7* showed strong predicted functional associations with several fibroblast growth factor receptors (*FGFRs*), including *FGFR2* (interaction score = 0.997), *FGFR1* (0.995), *FGFR4* (0.966), and *FGFR3* (0.863). Other highly connected proteins included *PDGFRA* (0.897), *KDR* (0.875), *HGF* (0.859), *PDGFRB* (0.855), *IGF1* (0.852), and *KIT* (0.841).

## 4. Discussion

In the current study, SNPs in four candidate genes (*FGF7*, *FGFR2*, *SMAD5*, and *SOSTDC1*) implicated in hair follicle development were evaluated for their associations with lambskin traits in Hu sheep. Among these genes, *FGF7* showed the most consistent evidence, with concordant associations across genotype and allele distributions, categorical lambskin phenotypes, and quantitative pattern-width analysis. *SMAD5* showed weaker nominal associations with selected traits, whereas *SOSTDC1* showed limited genotype-level evidence and *FGFR2* showed no significant SNP-level associations. Overall, the findings support the *FGF7* region as the strongest candidate signal identified in this study, while the other genes provide complementary evidence within interconnected follicle-development pathways.

The *PIC* values of the three *FGF7* SNPs were within the moderate polymorphism range, according to the classification of [34], indicating moderate genetic informativeness in the studied Hu sheep population. Observed heterozygosity (*He*) ranged from 0.4865 to 0.4995, with Chr7:57803771:C>T approaching the theoretical maximum of 0.50 for a biallelic marker. The corresponding effective number of alleles (*Ne*) was also close to the theoretical maximum of 2.0, consistent with a relatively balanced distribution of the two alleles at this locus. The observed *He* values were comparable with those reported for other candidate genes in Hu sheep, including *NR4A1* (0.37–0.48) and *FGF5* (0.35–0.45) [35]. Overall, the moderate *PIC* and relatively high heterozygosity observed at the *FGF7* loci indicate that these variants are sufficiently polymorphic for genetic association analyses in the studied population.

Most loci conformed to *HWE*, suggesting that these markers were not strongly affected by factors causing major departures from expected genotype frequencies, such as severe selection, population subdivision, or genotyping errors [32,36]. Similar *HWE* deviations have been reported at candidate loci associated with economically important traits in livestock populations [37,38]. However, such deviations alone do not establish artificial selection or linkage with a functional variant. For Chr7:57803771:C>T, representative genotypes were independently validated by Sanger sequencing, supporting the reliability of the genotyping results; however, because only representative samples were validated, genotyping error across the entire population cannot be completely excluded. The present dataset does not allow us to distinguish whether relatedness, population structure, selection, or other population processes contributed to the observed deviation. Therefore, although Chr7:57803771:C>T showed significant associations with lambskin traits after FDR correction, its deviation from *HWE* should be considered when interpreting the association. Further validation in independent Hu sheep populations, together with analyses of relatedness, population structure, linkage disequilibrium, and functional effects, will be necessary to establish the robustness and biological basis of this signal [37,38].

The most consistent evidence in the present study involved *FGF7*. The association of C-allele frequency at Chr7:57803771:C>T with pattern area and lambskin grade identified this locus as the most prominent marker. Two *FGF7* variants (Chr7:57741376:T>G and Chr7:57740946:G>A) showed similar allele-frequency patterns across wool-pattern categories and the mutant homozygous type (GG and AA) were associated with narrower patterns, whereas Chr7:57803771:C>T showed the opposite genotype-specific pattern, with the wild homozygous type (CC) associated with a wider pattern. The occurrence of multiple associated variants within the same genomic region strengthens the evidence for an *FGF7*-region signal. However, similar genotype distributions may reflect linkage disequilibrium among these markers, and their effects should therefore not be interpreted as independent without formal linkage-disequilibrium and conditional analyses.

The quantitative analysis provides complementary evidence for the association between the *FGF7* region and lambskin morphology. The agreement between the categorical analyses and the quantitative pattern-width results strengthens the interpretation that variation in the *FGF7* region is related to measurable differences in wool-pattern morphology. However, pattern width, pattern area, and overall lambskin grade represent related but distinct phenotypes. Pattern width describes a specific morphological characteristic, whereas lambskin grade integrates multiple aspects of commercial quality. Thus, an allele associated with pattern width does not necessarily have the same effect on overall lambskin grade, which may explain why Chr7:57803771:C>T was associated with both pattern area and grade, whereas the other *FGF7* variants were primarily associated with pattern width. This distinction is consistent with the polygenic architecture of economically important livestock traits, in which different loci may influence different components of a complex phenotype [39,40].

In a multi-trait QTL mapping study in sheep, *FGF7* was identified as a positional candidate within a genomic region associated with wool-production traits, highlighting its potential relevance to hair growth and follicular differentiation [41]. Ref. [42] also identified a polymorphism upstream of *FGF7* that contributed to divergence among sheep populations adapted to different altitudes. Subsequent analyses demonstrated that this regulatory variant is located in a highly conserved region and may influence transcription-factor binding and gene expression [43,44]. Although these studies examined different phenotypes from those investigated here, they provide important biological support for the responsiveness of the *FGF7* locus to genetic variation and environmental or phenotypic differentiation. Thus, the genotype-pattern relationship observed in Hu sheep is consistent with previous evidence that variation around *FGF7* can have functional consequences.

Developmental evidence from sheep further supports the potential involvement of *FGF7* in follicular biology. In Merino sheep, *FGF7* exhibits stage-specific expression during early hair follicle induction, including around embryonic day 65, when interactions between the epithelial placode and dermal condensate contribute to follicle formation [45]. Morphological and transcriptomic studies in Hu sheep have similarly demonstrated that differences in lambskin wave patterns are accompanied by changes in primary and secondary follicle characteristics and coordinated changes in follicle-related mRNA and microRNA expression [11,13,37,46]. These findings provide a plausible developmental link between genetic variation in follicle-related pathways and the morphological characteristics underlying wool-pattern variation.

Importantly, all significantly associated *FGF7* variants identified in this study were in non-coding regions: 3′UTR and 5′UTR. They provide a plausible regulatory explanation for the observed associations. Variants in the 3′UTR can influence mRNA stability, translation, or microRNA-mediated regulation, whereas 5′UTR variants may affect transcriptional regulation, translation initiation, RNA structure, or upstream regulatory elements [47,48,49]. Sheep studies have demonstrated that a 3′UTR insertion at the *IRF2BP2* locus was implicated in fleece variation following molecular characterization [50,51]. Therefore, the present *FGF7* SNPs may influence lambskin traits through regulatory effects, although linkage disequilibrium with an unobserved functional variant remains an equally plausible explanation.

The involvement of *FGF7* can also be considered in relation to its principal receptor, *FGFR2*. Disruption of *FGFR2* signaling has been shown to affect epithelial differentiation, hair-shaft structure, and follicular development [52,53]. Differences in *FGFR2* expression among fiber-producing species further suggest that the *FGF7*–*FGFR2* axis may contribute to differences in follicular characteristics [44]. The present absence of association with measured lambskin traits does not exclude a role for the pathway because the assayed variants may not be functional or may not be in linkage disequilibrium with functional variants. Thus, the present results provide stronger locus-specific support for *FGF7* than for *FGFR2*, while *FGFR2* remains biologically relevant as a component of the *FGF7* signaling pathway [54].

Evidence for *SMAD5* was weaker and less consistent than that for *FGF7*. The nominal association of Chr5:44907730:G>A with sacral wool length, and Chr5:44906657:G>A with categorical analyses, did not remain significant after FDR correction, providing suggestive rather than conclusive evidence for a contribution of *SMAD5* to lambskin traits. Recent genomic evidence identified *SMAD5* within runs of homozygosity associated with economically important traits in Kazakh fat-tailed coarse-wool sheep [55], while immunohistochemical analysis has demonstrated phosphorylated *SMAD5* expression in developing sheep skins [15]. Because BMP signaling contributes to the regulation of follicular stem-cell activity and the hair cycle [56,57], variation in *SMAD5* could plausibly influence follicular development or fiber elongation. The weaker statistical evidence for *SMAD5* compared with *FGF7* may reflect differences in effect size, allele frequency, linkage with causal variants, or the specific follicular process influenced by each gene. Therefore, *SMAD5* should be considered a biologically plausible contributor to wool-related variation rather than a major determinant based on the present dataset.

*SOSTDC1* provided additional but weaker biological support. The *SOSTDC1* variant investigated did not show a significant allele-frequency association, although its genotype distribution differed nominally among lambskin-grade categories. This observation is compatible with previous sheep studies implicating *SOSTDC1* in wool-type differentiation. In [58], using transcriptomic and whole-genome bisulfite sequencing approaches, the authors reported differential expression and methylation of *SOSTDC1* between wool types and localized its protein expression to primary wool follicles and dermal papilla. Similarly, network analysis identified *SOSTDC1* within a regulatory network involving genes associated with follicle morphogenesis, including *KRT25*, *BMP7*, *WNT10A*, and *TGFβ2* [37]. However, these previous studies primarily addressed gene expression and epigenetic regulation rather than the specific SNP examined here. Therefore, the nominal association observed in the present study should be interpreted as pathway-level evidence warranting further investigation rather than evidence that the SNP directly alters *SOSTDC1* function.

The PPI network constructed for *FGF7* provides a framework for interpreting its potential role in wool-follicle biology, although the interaction scores are computational predictions rather than experimentally demonstrated interactions in Hu sheep. The strongest predicted partner was *FGFR2* (score 0.997), consistent with the established role of *FGF7* as a keratinocyte growth factor that signals through the *FGFR2b* isoform to promote epithelial proliferation and hair follicle development [59]. The network also included *FGFR1*, *FGFR3* and *FGFR4*, consistent with *FGF7* acting within the broader fibroblast growth factor receptor family morphogenesis [20,60].

In addition to *FGFR2*, *FGF7* exhibited strong interactions with *PDGFRA* and *PDGFRB*. Members of the platelet-derived growth factor family are involved in dermal papilla development and mesenchymal cell proliferation, both of which are essential for normal hair follicle formation [61,62,63,64]. Furthermore, the interaction between *FGF7* and *KDR* (VEGFR2) suggests a possible role of angiogenesis in wool follicle development. VEGF signaling has been shown to regulate vascularization around hair follicles, thereby supporting follicular growth and cycling [65]. The predicted interactions with *HGF* and *IGF1* further support the involvement of *FGF7* in follicular growth and tissue regeneration. *HGF* promotes epithelial cell proliferation and morphogenesis, whereas *IGF1* regulates hair follicle development and prolongs the anagen phase of the hair cycle [16,66,67].

Several limitations should be considered when interpreting these findings. First, the candidate-gene association design cannot establish molecular causality, particularly for variants located in non-coding regions. Second, the sample size may have limited statistical power for rare genotype classes, as illustrated by the small number of animals carrying some genotypes. Third, the three associated *FGF7* variants may be correlated through linkage disequilibrium, making it difficult to determine whether they represent independent effects. Fourth, although partial parental information was available for some animals, complete pedigree information was not available for all 384 lambs, and some animals lacked recorded sire information. Therefore, the potential effects of family relatedness could not be fully assessed or accounted for in the present analysis and may have influenced the SNP–phenotype associations. Finally, the present analysis focused on associations with phenotypic traits and did not directly measure *FGF7* expression, allele-specific regulation, or molecular effects of the variants. Future studies should therefore include larger and independent Hu sheep populations with complete pedigree or genomic relatedness information, linkage-disequilibrium and conditional analyses, fine mapping, and functional validation in skin or dermal papilla tissues, including allele-specific expression, transcription-factor binding, and reporter assays.

## 5. Conclusions

This study identified significant associations between *FGF7* polymorphisms and lambskin traits in Hu sheep. After FDR correction, three *FGF7* SNPs remained significantly associated with wool-pattern characteristics, with Chr7:57803771:C>T showing the strongest association with fully wavy wool. The quantitative-trait analysis further supported the involvement of the *FGF7* region through associations with pattern width. Because the associated variants are in non-coding regions, they may influence lambskin phenotypes through regulatory mechanisms affecting *FGF7* expression or post-transcriptional and translational regulation, although this hypothesis requires functional validation. The PPI analysis provided additional pathway-level support for the biological relevance of *FGF7* in signaling networks involved in epithelial and follicular development. Collectively, these findings identify *FGF7* as a promising candidate locus underlying variation in wool-pattern and lambskin characteristics in Hu sheep. However, independent-population validation and functional experiments are required to determine whether the associated variants are causal and to establish their potential utility and function for Hu sheep breeding.

## Figures and Tables

**Table 1:** Summary of SNP distribution in candidate genes.

Gene	Chromosome	No. (SNP ID)	Genomic Region
*SOSTDC1*	Chr4	1 (SNP1)	5′UTR
*SMAD5*	Chr5	8 (SNP2–9)	5′UTR (SNP2–SNP5), 3′UTR (SNP6–SNP9)
*FGF7*	Chr7	3 (SNP10–12)	3′UTR (SNP10, SNP11), 5′UTR (SNP12)
*FGFR2*	Chr22	6 (SNP13–18)	3′UTR

**Note:** SNP names are shown in shortened form (Chr:Position:Ref>Alt). The reference genome is Oar_v4.0 (GCF_000298735.2). Full SNP identifiers are provided in Supplementary Table S1.

**Table 2:** Genetic diversity parameters of SNP loci in the studied sheep population.

Gene	SNP	N	Alleles	MAF	*H_o_*	*H_e_*	*N_e_*	*PIC*	HWE_ChiSq	HWE_P	PIC_Class
*FGF7*	Chr7:57740946:G>A	383	A/G	0.4178	0.4595	0.4865	1.9473	0.3681	1.1747	0.27844	Moderate
	Chr7:57741376:T>G	383	G/T	0.4178	0.4595	0.4865	1.9473	0.3681	1.1747	0.27844	Moderate
	Chr7:57803771:C>T	383	C/T	0.4843	0.3003	0.4995	1.998	0.3748	60.9397	*p* < 0.001	Moderate
*FGFR2*	Chr22:40345986:C>T	384	C/T	0.1445	0.237	0.2473	1.3285	0.2167	0.6668	0.414159	Low
	Chr22:40346188:T>C	383	C/T	0.3499	0.4648	0.4549	1.8346	0.3514	0.1788	0.672373	Moderate
	Chr22:40346469:G>C	383	C/G	0.4347	0.53	0.4915	1.9665	0.3707	2.356	0.124799	Moderate
	Chr22:40346646:C>T	384	C/T	0.4557	0.5156	0.4961	1.9844	0.373	0.5961	0.440085	Moderate
	Chr22:40347084:G>A	383	A/G	0.4948	0.5196	0.4999	1.9998	0.375	0.5909	0.442082	Moderate
	Chr22:40348212:A>G	384	A/G	0.319	0.4661	0.4345	1.7683	0.3401	2.039	0.153314	Moderate
*SMAD5*	Chr5:44905960:T>G	382	G/T	0.4817	0.4607	0.4993	1.9973	0.3747	2.2822	0.130862	Moderate
	Chr5:44906216:C>T	383	C/T	0.248	0.3708	0.373	1.595	0.3035	0.0143	0.904916	Moderate
	Chr5:44906657:G>A	383	A/G	0.1188	0.2219	0.2094	1.2648	0.1875	1.3784	0.240369	Low
	Chr5:44907171:T>G	384	G/T	0.1758	0.2891	0.2898	1.408	0.2478	0.0023	0.96214	Low
	Chr5:44907730:G>A	383	A/G	0.4295	0.5039	0.4901	1.961	0.37	0.3062	0.580039	Moderate
	Chr5:44908255:A>G	384	A/G	0.0794	0.1276	0.1462	1.1713	0.1355	6.234	0.012532	Low
	Chr5:44852712:A>T	382	A/T	0.2945	0.4162	0.4155	1.711	0.3292	0.001	0.974158	Moderate
	Chr5:44852977:T>A	382	A/T	0.4424	0.5288	0.4934	1.9738	0.3717	1.9699	0.160455	Moderate
*SOSTDC1*	Chr4:25022929:A>T	383	A/T	0.2742	0.3708	0.398	1.6611	0.3188	1.7926	0.180608	Moderate

Note: MAF, minor allele frequency; *H_o_*, observed heterozygosity; *H_e_*, expected heterozygosity; *N_e_*, effective number of alleles; *PIC*, polymorphism information content; HWE, Hardy–Weinberg equilibrium. *p* < 0.05 indicates significant deviation from HWE.SNP names are shown in shortened form (Chr:Position:Ref>Alt). The reference genome is Oar_v4.0 (GCF_000298735.2). Full SNP identifiers are provided in Supplementary Tables S1–S5.

**Table 3:** Allele frequencies of *FGF7* and *SMAD5* variants across pattern area and grade categories in Hu sheep.

Name	Allele	Pattern Area					Lambskin Grade				
		0/4	1/4	2/4	3/4	4/4	1	2	3	4	5
*FGF7* (Chr7:57803771:C>T)	C	0.30 (22)	0.42 (38)	0.40 (32)	0.54 (57)	0.53 (222)	0.30 (22)	0.42 (38)	0.54 (98)	0.49 (112)	0.53 (101)
	T	0.70 (52)	0.58 (52)	0.60 (48)	0.46 (49)	0.47 (194)	0.70 (52)	0.58 (52)	0.46 (82)	0.51 (118)	0.47 (91)
	χ^2^			19.30					15.70		
	*p*			0.001					0.003		
*FGF7* (Chr7:57740946:G>A)	A	0.54 (40)	0.50 (44)	0.54 (43)	0.42 (45)	0.35 (148)	0.54 (40)	0.50 (44)	0.36 (65)	0.41 (96)	0.39 (75)
	G	0.46 (34)	0.50 (44)	0.46 (37)	0.58 (61)	0.65 (270)	0.46 (34)	0.50 (44)	0.64 (115)	0.59 (136)	0.61 (117)
	χ^2^			18.74					10.00		
	*p*			0.001					0.040		
*FGF7* (Chr7:57741376:T>G)	G	0.54 (40)	0.50 (45)	0.54 (42)	0.42 (45)	0.35 (148)	0.54 (40)	0.50 (45)	0.36 (65)	0.41 (95)	0.39 (75)
	T	0.46 (34)	0.50 (45)	0.46 (36)	0.58 (61)	0.65 (270)	0.46 (34)	0.50 (45)	0.64 (115)	0.59 (135)	0.61 (117)
	χ^2^			18.75					10.07		
	*p*			0.001					0.039		
*SMAD5* (Chr5:44852712:A>T)	A	0.65 (48)	0.82 (74)	0.69 (54)	0.66 (70)	0.70 (293)	0.65 (48)	0.82 (74)	0.66 (118)	0.74 (170)	0.67 (129)
	T	0.35 (26)	0.18 (16)	0.31 (24)	0.34 (36)	0.30 (123)	0.35 (26)	0.18 (16)	0.34 (62)	0.26 (58)	0.33 (63)
	χ^2^			8.16					12.02		
	*p*			0.086					0.017		
*SMAD5* (Chr5:44906657:G>A)	G	0.80 (59)	0.93 (84)	0.90 (70)	0.87 (92)	0.89 (370)	0.80 (59)	0.93 (84)	0.87 (157)	0.91 (209)	0.86 (166)
	A	0.20 (15)	0.07 (6)	0.10 (8)	0.13 (14)	0.11 (48)	0.20 (15)	0.07 (6)	0.13 (23)	0.09 (21)	0.14 (26)
	χ^2^			7.75					9.62		
	*p*			0.101					0.047		

Note: Values are presented as allele frequency (allele count). The value before the parentheses represents the allele frequency, and the value in parentheses represents the corresponding allele count. Pattern-area categories: 0/4 = straight wool; 1/4 = 25% wavy; 2/4 = 50% wavy; 3/4 = 75% wavy; 4/4 = fully wavy. Lambskin grade: 1 = highest quality; 2–5 = progressively lower quality. SNP names are shown in shortened form (Chr:Position:Ref>Alt). The reference genome is Oar_v4.0 (GCF_000298735.2). Full SNP identifiers are provided in Supplementary Table S2.

**Table 4:** Significant allelic associations with odds ratios and FDR correction.

Gene	SNP	Trait	χ^2^ (df = 4)	*P*	FDR q	OR	95% CI
*FGF7*	Chr7:57803771:C>T	pattern area	19.30	0.00069	0.011	2.70	1.59–4.62
	Chr7:57803771:C>T	lambskin grade	15.70	0.00344	0.031	2.62	1.48–4.65
	Chr7:57740946:G>A	pattern area	18.74	0.00088	0.011	0.47	0.28–0.77
	Chr7:57741376:T>G	pattern area	18.75	0.00088	0.011	0.47	0.28–0.77
*SMAD5*	Chr5:44852712:A>T	lambskin grade	12.02	0.01717	0.124	1.11	0.63–1.95
	Chr5:44906657:G>A	lambskin grade	9.62	0.04736	0.213	0.62	0.31–1.24

Note: FDR, false discovery rate; OR, odds ratio; CI, confidence interval. FDR-adjusted q < 0.05 was considered significant. SNP names are shown in shortened form. Full identifiers (Oar_v4.0:Chr:Position:Ref>Alt) are provided in Supplementary Table S1.

**Table 5:** Genotype frequencies of *FGF7* and *SOSTDC1* variants across pattern area and grade categories in Hu sheep.

Name	Genotype	Pattern Type					Lambskin Grade				
		0/4	1/4	2/4	3/4	4/4	1	2	3	4	5
*FGF7* (Chr7: 57741376:T>G)	GG	0.32 (12)	0.24 (11)	0.31 (12)	0.15 (8)	0.14 (29)	0.32 (12)	0.24 (11)	0.18 (16)	0.17 (20)	0.14 (13)
	GT	0.43 (16)	0.51 (23)	0.46 (18)	0.55 (29)	0.43 (90)	0.43 (16)	0.51 (23)	0.37 (33)	0.48 (55)	0.51 (49)
	TT	0.24 (9)	0.24 (11)	0.23 (9)	0.30 (16)	0.43 (90)	0.24 (9)	0.24 (11)	0.46 (41)	0.35 (40)	0.35 (34)
	χ^2^			20.449					14.105		
	*p*			0.0086					0.0791		
*FGF7* (Chr7: 57740946:G>A)	AA	0.32 (12)	0.25 (11)	0.30 (12)	0.15 (8)	0.14 (29)	0.32 (12)	0.24 (11)	0.18 (16)	0.17 (20)	0.14 (13)
	AG	0.43 (16)	0.50 (22)	0.48 (19)	0.55 (29)	0.43 (90)	0.43 (16)	0.50 (22)	0.37 (33)	0.48 (56)	0.51 (49)
	GG	0.24 (9)	0.25 (11)	0.23 (9)	0.30 (16)	0.43 (90)	0.24 (9)	0.24 (11)	0.46 (41)	0.34 (40)	0.35 (34)
	χ^2^			20.226					14.048		
	*p*			0.0097					0.0808		
*FGF7* (Chr7: 57803771:C>T)	CC	0.11 (4)	0.29 (13)	0.35 (14)	0.42 (22)	0.36 (75)	0.11 (4)	0.29 (13)	0.42 (38)	0.35 (40)	0.34 (33)
	CT	0.38 (14)	0.27 (12)	0.10 (4)	0.25 (13)	0.35 (72)	0.38 (14)	0.27 (12)	0.24 (22)	0.28 (32)	0.36 (35)
	TT	0.51 (19)	0.44 (20)	0.55 (22)	0.34 (18)	0.29 (61)	0.51 (19)	0.44 (20)	0.33 (30)	0.37 (43)	0.29 (28)
	χ^2^			25.468					16.169		
	*p*			0.0012					0.0401		
*SOSTDC1* (Chr4: 25022929:A>T)	AA	0.51 (19)	0.53 (24)	0.50 (20)	0.53 (28)	0.56 (116)	0.51 (19)	0.53 (24)	0.48 (43)	0.61 (70)	0.53 (51)
	AT	0.30 (11)	0.29 (13)	0.48 (19)	0.40 (21)	0.38 (78)	0.30 (11)	0.29 (13)	0.46 (41)	0.33 (38)	0.41 (39)
	TT	0.19 (7)	0.18 (8)	0.03 (1)	0.08 (4)	0.07 (14)	0.19 (7)	0.18 (8)	0.07 (6)	0.06 (7)	0.06 (6)
	χ^2^			14.167					16.111		
	*p*			0.0778					0.0410		

Note: Numbers in parentheses indicate the count of alleles. See Table 3 for definitions of the pattern type and lambskin grade categories. SNP names are shown in shortened form (Chr:Position:Ref>Alt). The reference genome is Oar_v4.0 (GCF_000298735.2). Full SNP identifiers are provided in Supplementary Table S4.

**Table 6:** Associations between SNP genotypes and quantitative lambskin traits in Hu sheep.

Gene	SNP	Genotype	N	Mean ± SD	Sig.
		Sacral wool length			
*SMAD5*	Chr5:44907730:G>A	AA	122	2.87 ± 0.66 ^b^	0.0152
		AG	193	2.79 ± 0.63 ^b^	
		GG	68	3.06 ± 0.71 ^a^	
		Pattern width			
*FGF7*	Chr7:57740946:G>A	AA	72	1.23 ± 0.73 ^b^	0.0021
		AG	176	1.51 ± 0.65 ^a^	
		GG	135	1.55 ± 0.57 ^a^	
*FGF7*	Chr7:57741376:T>G	GG	72	1.24 ± 0.73 ^b^	0.0022
		GT	176	1.52 ± 0.65 ^a^	
		TT	135	1.55 ± 0.57 ^a^	
*FGF7*	Chr7:57803771:C>T	CC	128	1.62 ± 0.49 ^a^	0.0039
		CT	115	1.46 ± 0.67 ^ab^	
		TT	140	1.36 ± 0.73 ^b^	
*SMAD5*	Chr5:44906657:G>A	AA	3	0.83 ± 0.72 ^c^	0.0229
		AG	85	1.34 ± 0.67 ^b^	
		GG	295	1.52 ± 0.63 ^a^	

Note: Different lowercase letters (a, b, c) within each trait and SNP indicate significant differences by Tukey’s HSD post-hoc test (*p* < 0.05). SNP names are shown in shortened form (Chr:Position:Ref>Alt). The reference genome is Oar_v4.0 (GCF_000298735.2). Full SNP identifiers are provided in Supplementary Table S5.

**Table 7:** Major proteins interacting with *FGF7* predicted by STRING.

Protein	Full Name	Function	Score
*FGFR2*	Fibroblast growth factor receptor 2	Primary receptor of *FGF7* regulating keratinocyte proliferation and hair follicle development	0.997
*FGFR1*	Fibroblast growth factor receptor 1	Mediates FGF signaling and epithelial cell proliferation	0.995
*FGFR4*	Fibroblast growth factor receptor 4	Participates in cell growth and metabolic signaling	0.966
*PDGFRA*	Platelet-derived growth factor receptor alpha	Regulates cell proliferation and dermal development	0.897
*KDR*	Kinase insert domain receptor (VEGFR2)	Controls angiogenesis and vascular signaling in skin tissue	0.875
*FGFR3*	Fibroblast growth factor receptor 3	Regulates tissue growth and differentiation	0.863
*HGF*	Hepatocyte growth factor	Stimulates epithelial cell growth and morphogenesis	0.859
*PDGFRB*	Platelet-derived growth factor receptor beta	Involved in vascular and follicle development	0.855
*IGF1*	Insulin-like growth factor 1	Promotes cell growth and follicular development	0.852
*KIT*	KIT proto-oncogene receptor tyrosine kinase	Regulates cell survival and follicle stem cell activity	0.841

## Data Availability

The raw data supporting the conclusions of this article will be made available by the authors upon request.

## Supplementary Materials

The following supporting information can be downloaded at: https://www.mdpi.com/article/10.3390/ani16193126/s1. Supplementary Table S1. Detailed information for all SNP loci analyzed in this study, including the full SNP identifier (Oar_v4.0:Chr:Position:Ref>Alt), gene name, reference genome, chromosome, genomic position, allele variation, and genomic region; Supplementary Table S2. Primer sequences for the 18 SNP loci in *SOSTDC1*, *SMAD5*, *FGF7*, and *FGFR2*; Supplementary Table S3. Allele frequencies of all polymorphic SNPs across pattern-area and grade categories; Supplementary Table S4. Complete genotypic frequency table for all polymorphic SNPs; Supplementary Table S5. Associations between SNP genotypes and quantitative lambskin traits in Hu sheep.
