# Supplementary material for: FGF7 Polymorphisms, Especially Chr7:57803771:C>T, are Associated with Lambskin Traits in Hu Sheep
Source: Animals (Basel). 2026 Oct 5;16(19):3126. doi: 10.3390/ani16193126 (PMC13663528; doi:10.3390/ani16193126)
Supplement: Supplementary file 1 [file animals-16-03126-s001.zip › Supplementary Table S1.pdf]

Supplementary Table S1. Detailed information of all SNP loci analyzed in this study, including original SNP identifier, gene name, chromosome, genomic position in Chr:g. format, allele variation, and genomic region.

SNP_ID	Gene	Chromosome	Genomic Position	Allele (Ref/Alt)	Genomic Region
SNP1	*SOSTDC1*	Chr4	Oar_v4.0:Chr4:25022929:A>T	A/T	5′UTR
SNP2	*SMAD5*	Chr5	Oar_v4.0:Chr5:44852712:A>T	A/T	5′UTR
SNP3	*SMAD5*	Chr5	Oar_v4.0:Chr5:44852977:T>A	T/A	5′UTR
SNP4	*SMAD5*	Chr5	Oar_v4.0:Chr5:44905960:T>G	T/G	5′UTR
SNP5	*SMAD5*	Chr5	Oar_v4.0:Chr5:44906216:C>T	C/T	5′UTR
SNP6	*SMAD5*	Chr5	Oar_v4.0:Chr5:44906657:G>A	G/A	3′UTR
SNP7	*SMAD5*	Chr5	Oar_v4.0:Chr5:44907171:T>G	T/G	3′UTR
SNP8	*SMAD5*	Chr5	Oar_v4.0:Chr5:44907730:G>A	G/A	3′UTR
SNP9	*SMAD5*	Chr5	Oar_v4.0:Chr5:44908255:A>G	A/G	3′UTR
SNP10	*FGF7*	Chr7	Oar_v4.0:Chr7:57740946:G>A	G/A	3′UTR
SNP11	*FGF7*	Chr7	Oar_v4.0:Chr7:57741376:T>G	T/G	3′UTR
SNP12	*FGF7*	Chr7	Oar_v4.0:Chr7:57803771:C>T	C/T	5′UTR
SNP13	*FGFR2*	Chr22	Oar_v4.0:Chr22:40345986:C>T	C/T	3′UTR
SNP14	*FGFR2*	Chr22	Oar_v4.0:Chr22:40346188:T>C	T/C	3′UTR
SNP15	*FGFR2*	Chr22	Oar_v4.0:Chr22:40346469:G>C	G/C	3′UTR
SNP16	*FGFR2*	Chr22	Oar_v4.0:Chr22:40346646:C>T	C/T	3′UTR
SNP17	*FGFR2*	Chr22	Oar_v4.0:Chr22:40347084:G>A	G/A	3′UTR
SNP18	*FGFR2*	Chr22	Oar_v4.0:Chr22:40348212:A>G	A/G	3′UTR
