# Supplementary material for: FGF7 Polymorphisms, Especially Chr7:57803771:C>T, are Associated with Lambskin Traits in Hu Sheep
Source: Animals (Basel). 2026 Oct 5;16(19):3126. doi: 10.3390/ani16193126 (PMC13663528; doi:10.3390/ani16193126)
Supplement: Supplementary file 1 [file animals-16-03126-s001.zip › Supplementary Table S2.pdf]

Supplementary Table S2. Primer sequences for the 18 SNP loci in *SOSTDC1*, *SMAD5*, *FGF7*, and *FGFR2*

Gene	SNP locus	Forward primer (5′–3′)	Reverse primer (5′–3′)	Extension primer (5′–3′)
SOSTDC1	Oar_v4.0:Chr4:25022929:A>T	ACGTTGGATGCCCCTCCCTACTACAAATAC	ACGTTGGATGCTAGTTCACATTCGGGTTTC	AATATGCTGCTGTTC
SMAD5	Oar_v4.0:Chr5:44852712:A>T	ACGTTGGATGCTCTACATACTACCGGTAAC	ACGTTGGATGCCTTCTAAAAATGTCTTGG	gggcgTGGTAACTTGAAATACTATTT
SMAD5	Oar_v4.0:Chr5:44852977:T>A	ACGTTGGATGGTAGTCTGTTCGTGTGCTTC	ACGTTGGATGACCCACAGGATATAAGGGAG	aagAAACTATTTCAGTTCTTC
SMAD5	Oar_v4.0:Chr5:44905960:T>G	ACGTTGGATGATTAGGTGATTTCCCACCCG	ACGTTGGATGAGGGAAGGACTCATAACCTC	ACTCATAACCTCTTTTCC
SMAD5	Oar_v4.0:Chr5:44906216:C>T	ACGTTGGATGGGCACTGCATCTGAGAAAAC	ACGTTGGATGTTGAGGAAAGGAGAATGCAG	ccccCAGTTTTTTTATTTTCCGTAA
SMAD5	Oar_v4.0:Chr5:44906657:G>A	ACGTTGGATGCATTCATGAGACACAACTCC	ACGTTGGATGCATGTAGCAACCTTTAGAG	gttgATGTAATTGGCTTTTTCT
SMAD5	Oar_v4.0:Chr5:44907171:T>G	ACGTTGGATGGCACCTCTTAAATTCTGCTG	ACGTTGGATGGGTAAATGCAAAAAAGAGGC	tgAAAGCAACAAAAATATACTGACT
SMAD5	Oar_v4.0:Chr5:44907730:G>A	ACGTTGGATGCTGCAACAGAAGTACAACAC	ACGTTGGATGATTCCCCTGTCATAGTGTTC	ggaCAGTAACAGAGAAACTATAA
SMAD5	Oar_v4.0:Chr5:44908255:A>G	ACGTTGGATGTTGATGCCTATCTCCCTCAG	ACGTTGGATGCTCTCCACACATGCATCTTC	agaAGAGCCCCACCTGTGTTAG
FGFR2	Oar_v4.0:Chr22:40345986:C>T	ACGTTGGATGGCCAGAACTCAACTTTTCAG	ACGTTGGATGGCTCCACTAATAGCCTCAAC	gggTTTTCAAGAGAAATATTTTA
FGFR2	Oar_v4.0:Chr22:40346188:T>C	ACGTTGGATGGGCTGGCAAGTGATCTAATG	ACGTTGGATGTGTACTGTTTCCATGCCACC	ttcccTGCCACCAAGAATCTG
FGFR2	Oar_v4.0:Chr22:40346469:G>C	ACGTTGGATGATGCCCTTCATTTGCTCCTC	ACGTTGGATGGAAAAGGCTGTTCAAGTCC	cccCCTGGCTTCGTTGCTTC
FGFR2	Oar_v4.0:Chr22:40346646:C>T	ACGTTGGATGGTAGCAAACAACAGACCTCC	ACGTTGGATGGTCTTACCAAGTGGTTTCCC	gtgTCCCGTGCTCGGAGGC
FGFR2	Oar_v4.0:Chr22:40347084:G>A	ACGTTGGATGAGCCTGTATTCTCTTTGGCG	ACGTTGGATGCAATGTTTCCACTTTCTCCG	ttcgAAGAGACTTCTCTGTTGCCA
FGFR2	Oar_v4.0:Chr22:40348212:A>G	ACGTTGGATGGCTCAGTACAGCTAAGTTCC	ACGTTGGATGCAGTATCCACATAGAAACGG	gACATGAATGGGCCTGTCC
FGF7	Oar_v4.0:Chr7:57740946:G>A	ACGTTGGATGTACCCTTTCAATTGGAGCAG	ACGTTGGATGATTCCATCATGGAAATGAG	GGAAATGAGTCAAAG
FGF7	Oar_v4.0:Chr7:57741376:T>G	ACGTTGGATGCTTTCTTTAATGCAGTCTC	ACGTTGGATGCCAAGAAAGGCTGGAAAAC	gtggTCAAGCTGGACTTGCG
FGF7	Oar_v4.0:Chr7:57803771:C>T	ACGTTGGATGAGATATCCCCTTAACTCAGC	ACGTTGGATGATGAAGCGTACATTGCTAC	agctTACATTGCTACTATTATTCCT

Note: F, forward primer; R, reverse primer; EXT, extension primer (UEP). Lowercase letters in extension-primer sequences indicate non-template sequences added for mass adjustment, where applicable. For each SNP, the EXT sequence represents the common UEP sequence used for allele-specific extension.
