# Supplementary material for: FGF7 Polymorphisms, Especially Chr7:57803771:C>T, are Associated with Lambskin Traits in Hu Sheep
Source: Animals (Basel). 2026 Oct 5;16(19):3126. doi: 10.3390/ani16193126 (PMC13663528; doi:10.3390/ani16193126)
Supplement: Supplementary file 1 [file animals-16-03126-s001.zip › Supplementary Table S3.pdf]

Supplementary Table S3. Allele frequencies of all polymorphic SNPs across pattern area and grade categories

Name	Allele	Pattern area					Lambskin grade					χ^2 (Pattern)	P (Pattern)	χ^2 (Grade)	P (Grade)
		0/4	1/4	2/4	3/4	4/4	Grade_1	Grade_2	Grade_3	Grade_4	Grade_5				
SOSTDC1 (Oar_v4.0:Chr4:25022929:A>T)	A	0.66 (49)	0.68 (61)	0.74 (59)	0.73 (77)	0.75 (310)	0.66 (49)	0.68 (61)	0.71 (127)	0.77 (178)	0.73 (141)	3.39	0.495	5.67	0.225
	T	0.34 (25)	0.32 (29)	0.26 (21)	0.27 (29)	0.25 (106)	0.34 (25)	0.32 (29)	0.29 (53)	0.23 (52)	0.27 (51)				
SMAD5 (Oar_v4.0:Chr5:44852712:A>T)	A	0.65 (48)	0.82 (74)	0.69 (54)	0.66 (70)	0.70 (293)	0.65 (48)	0.82 (74)	0.66 (118)	0.74 (170)	0.67 (129)	8.16	0.086	12.02	0.017
	T	0.35 (26)	0.18 (16)	0.31 (24)	0.34 (36)	0.30 (123)	0.35 (26)	0.18 (16)	0.34 (62)	0.26 (58)	0.33 (63)				
SMAD5 (Oar_v4.0:Chr5:44852977:T>A)	T	0.84 (62)	0.80 (72)	0.74 (58)	0.75 (79)	0.73 (305)	0.84 (62)	0.80 (72)	0.69 (125)	0.75 (172)	0.76 (145)	5.21	0.267	7.26	0.123
	A	0.16 (12)	0.20 (18)	0.26 (20)	0.25 (27)	0.27 (113)	0.16 (12)	0.20 (18)	0.31 (55)	0.25 (58)	0.24 (47)				
SMAD5 (Oar_v4.0:Chr5:44905960:T>G)	T	0.49 (36)	0.61 (55)	0.51 (41)	0.52 (55)	0.50 (209)	0.49 (36)	0.61 (55)	0.47 (85)	0.55 (126)	0.49 (94)	3.72	0.446	6.16	0.187
	G	0.51 (38)	0.39 (35)	0.49 (39)	0.48 (51)	0.50 (207)	0.51 (38)	0.39 (35)	0.53 (95)	0.45 (104)	0.51 (96)				
SMAD5 (Oar_v4.0:Chr5:44906216:C>T)	C	0.84 (62)	0.80 (72)	0.74 (58)	0.75 (79)	0.73 (305)	0.84 (62)	0.80 (72)	0.69 (125)	0.75 (172)	0.76 (145)	5.21	0.267	7.26	0.123
	T	0.16 (12)	0.20 (18)	0.26 (20)	0.25 (27)	0.27 (113)	0.16 (12)	0.20 (18)	0.31 (55)	0.25 (58)	0.24 (47)				
SMAD5 (Oar_v4.0:Chr5:44906657:G>A)	G	0.80 (59)	0.93 (84)	0.90 (70)	0.87 (92)	0.89 (370)	0.80 (59)	0.93 (84)	0.87 (157)	0.91 (209)	0.86 (166)	7.75	0.101	9.62	0.047
	A	0.20	0.07	0.10	0.13	0.11	0.20 (15)	0.07 (6)	0.13 (23)	0.09 (21)	0.14 (26)				

		(15)	(6)	(8)	(14)	(48)									
SMAD5 (Oar_v4.0:Chr5:44907171:T>G)	T	0.84 (62)	0.80 (72)	0.80 (64)	0.79 (84)	0.84 (351)	0.84 (62)	0.80 (72)	0.86 (155)	0.79 (184)	0.83 (160)	2.21	0.696	3.81	0.432
	G	0.16 (12)	0.20 (18)	0.20 (16)	0.21 (22)	0.16 (67)	0.16 (12)	0.20 (18)	0.14 (25)	0.21 (48)	0.17 (32)				
SMAD5 (Oar_v4.0:Chr5:44907730:G>A)	G	0.60 (43)	0.63 (57)	0.58 (46)	0.60 (64)	0.54 (227)	0.60 (43)	0.63 (57)	0.48 (87)	0.61 (142)	0.56 (108)	3.43	0.489	8.93	0.063
	A	0.40 (29)	0.37 (33)	0.42 (34)	0.40 (42)	0.46 (191)	0.40 (29)	0.37 (33)	0.52 (93)	0.39 (90)	0.44 (84)				
SMAD5 (Oar_v4.0:Chr5:44908255:A>G)	A	0.93 (69)	0.88 (79)	0.90 (72)	0.91 (96)	0.94 (391)	0.93 (69)	0.88 (79)	0.91 (163)	0.93 (215)	0.94 (181)	4.44	0.350	4.36	0.360
	G	0.07 (5)	0.12 (11)	0.10 (8)	0.09 (10)	0.06 (27)	0.07 (5)	0.12 (11)	0.09 (17)	0.07 (17)	0.06 (11)				
FGF7 (Oar_v4.0:Chr7:57740946:G>A)	A	0.54 (40)	0.50 (44)	0.54 (43)	0.42 (45)	0.35 (148)	0.54 (40)	0.50 (44)	0.36 (65)	0.41 (96)	0.39 (75)	18.74	0.001	10.00	0.040
	G	0.46 (34)	0.50 (44)	0.46 (37)	0.58 (61)	0.65 (270)	0.46 (34)	0.50 (44)	0.64 (115)	0.59 (136)	0.61 (117)				
FGF7 (Oar_v4.0:Chr7:57741376:T>G)	G	0.54 (40)	0.50 (45)	0.54 (42)	0.42 (45)	0.35 (148)	0.54 (40)	0.50 (45)	0.36 (65)	0.41 (95)	0.39 (75)	18.75	0.001	10.07	0.039
	T	0.46 (34)	0.50 (45)	0.46 (36)	0.58 (61)	0.65 (270)	0.46 (34)	0.50 (45)	0.64 (115)	0.59 (135)	0.61 (117)				
FGF7 (Oar_v4.0:Chr7:57803771:C>T)	C	0.30 (22)	0.42 (38)	0.40 (32)	0.54 (57)	0.53 (222)	0.30 (22)	0.42 (38)	0.54 (98)	0.49 (112)	0.53 (101)	19.30	0.001	15.70	0.003
	T	0.70 (52)	0.58 (52)	0.60 (48)	0.46 (49)	0.47 (194)	0.70 (52)	0.58 (52)	0.46 (82)	0.51 (118)	0.47 (91)				
FGFR2 (Oar_v4.0:Chr22:40345986:C>T)	C	0.88 (65)	0.83 (75)	0.81 (65)	0.90 (95)	0.85 (357)	0.88 (65)	0.83 (75)	0.89 (160)	0.85 (196)	0.84 (161)	3.30	0.510	2.95	0.566
	T	0.12	0.17	0.19	0.10	0.15	0.12 (9)	0.17 (15)	0.11 (20)	0.15 (36)	0.16 (31)				

		(9)	(15)	(15)	(11)	(61)									
FGFR2 (Oar_v4.0:Chr22:40346188:T>C)	T	0.31 (23)	0.38 (34)	0.33 (26)	0.34 (36)	0.36 (149)	0.31 (23)	0.38 (34)	0.38 (68)	0.35 (81)	0.32 (62)	1.03	0.906	2.04	0.729
	C	0.69 (51)	0.62 (56)	0.67 (52)	0.66 (70)	0.64 (269)	0.69 (51)	0.62 (56)	0.62 (112)	0.65 (149)	0.68 (130)				
FGFR2 (Oar_v4.0:Chr22:40346469:G>C)	G	0.59 (44)	0.53 (47)	0.60 (48)	0.61 (65)	0.55 (229)	0.59 (44)	0.53 (47)	0.58 (104)	0.56 (131)	0.56 (107)	2.51	0.643	0.77	0.942
	C	0.41 (30)	0.47 (41)	0.40 (32)	0.39 (41)	0.45 (189)	0.41 (30)	0.47 (41)	0.42 (76)	0.44 (101)	0.44 (85)				
FGFR2 (Oar_v4.0:Chr22:40346646:C>T)	C	0.47 (35)	0.60 (54)	0.56 (45)	0.49 (52)	0.56 (232)	0.47 (35)	0.60 (54)	0.53 (95)	0.54 (125)	0.57 (109)	4.18	0.382	3.29	0.510
	T	0.53 (39)	0.40 (36)	0.44 (35)	0.51 (54)	0.44 (186)	0.53 (39)	0.40 (36)	0.47 (85)	0.46 (107)	0.43 (83)				
FGFR2 (Oar_v4.0:Chr22:40347084:G>A)	G	0.57 (42)	0.44 (40)	0.46 (36)	0.54 (57)	0.49 (204)	0.57 (42)	0.44 (40)	0.49 (89)	0.48 (110)	0.51 (98)	3.68	0.450	2.92	0.571
	A	0.43 (32)	0.56 (50)	0.54 (42)	0.46 (49)	0.51 (214)	0.43 (32)	0.56 (50)	0.51 (91)	0.52 (120)	0.49 (94)				
FGFR2 (Oar_v4.0:Chr22:40348212:A>G)	A	0.70 (52)	0.66 (59)	0.70 (56)	0.69 (73)	0.68 (283)	0.70 (52)	0.66 (59)	0.67 (120)	0.69 (159)	0.69 (133)	0.62	0.961	0.74	0.946
	G	0.30 (22)	0.34 (31)	0.30 (24)	0.31 (33)	0.32 (135)	0.30 (22)	0.34 (31)	0.33 (60)	0.31 (73)	0.31 (59)				

Notes:

1.Values are presented as frequency (count). Percentages are calculated within each phenotype category (column percentage).

2. Total sample size for Pattern area: 0/4 (n=74), 1/4 (n=88-90), 2/4 (n=78-80), 3/4 (n=106), 4/4 (n=416-418).

3. Total sample size for Lambskin grade: Grade_1 (n=74), Grade_2 (n=88-90), Grade_3 (n=180), Grade_4 (n=230-232), Grade_5 (n=192).

4. χ^2 values are Pearson Chi-square statistics with df = 4 (2 alleles × 5 categories).

6. Monomorphic SNPs (SNP2, SNP4, SNP29) were excluded from analysis.
