# Supplementary material for: FGF7 Polymorphisms, Especially Chr7:57803771:C>T, are Associated with Lambskin Traits in Hu Sheep
Source: Animals (Basel). 2026 Oct 5;16(19):3126. doi: 10.3390/ani16193126 (PMC13663528; doi:10.3390/ani16193126)
Supplement: Supplementary file 1 [file animals-16-03126-s001.zip › Supplementary Table S4.pdf]

Supplementary Table S4. Complete Genotypic Frequency Table for All Polymorphic SNPs

Name	Genotype	Pattern type					Lambskin grade					χ^2 (Pattern)	P (Pattern)	χ^2 (Grade)	P (Grade)
		0/4	1/4	2/4	3/4	4/4	1	2	3	4	5				
SOSTDC1 (Oar_v4.0:Chr4:25022929:A>T)	AA	0.51	0.53	0.50	0.53	0.56	0.51	0.53	0.48	0.61	0.53				
		(19)	(24)	(20)	(28)	(116)	(19)	(24)	(43)	(70)	(51)				
	AT	0.30	0.29	0.48	0.40	0.38	0.30	0.29	0.46	0.33	0.41	14.167	0.078	16.111	0.041*
		(11)	(13)	(19)	(21)	(78)	(11)	(13)	(41)	(38)	(39)				
	TT	0.19	0.18	0.03	0.08	0.07	0.19	0.18	0.07	0.06	0.06				
		(7)	(8)	(1)	(4)	(14)	(7)	(8)	(6)	(7)	(6)				
SMAD5 (Oar_v4.0:Chr5:44852712:A>T)	AA	0.24	0.31	0.23	0.19	0.15	0.24	0.31	0.18	0.22	0.19				
		(9)	(14)	(9)	(10)	(32)	(9)	(14)	(16)	(25)	(18)				
	AT	0.32	0.56	0.49	0.45	0.45	0.32	0.56	0.49	0.51	0.53	10.383	0.239	13.200	0.105
		(12)	(25)	(19)	(24)	(93)	(12)	(25)	(44)	(58)	(51)				
	TT	0.43	0.13	0.28	0.36	0.39	0.43	0.13	0.33	0.27	0.28				
		(16)	(6)	(11)	(19)	(82)	(16)	(6)	(30)	(31)	(27)				
SMAD5 (Oar_v4.0:Chr5:44852977:T>A)	TT	0.70	0.62	0.56	0.57	0.53	0.70	0.62	0.49	0.59	0.54				
		(26)	(28)	(22)	(30)	(111)	(26)	(28)	(44)	(68)	(52)				
	TA	0.27	0.36	0.36	0.36	0.40	0.27	0.36	0.43	0.31	0.43	5.851	0.664	13.200	0.105
		(10)	(16)	(14)	(19)	(83)	(10)	(16)	(39)	(36)	(41)				
	AA	0.03	0.02	0.08	0.08	0.07	0.03	0.02	0.08	0.10	0.03				
		(1)	(1)	(3)	(4)	(15)	(1)	(1)	(7)	(11)	(3)				
SMAD5 (Oar_v4.0:Chr5:44906657:G>A)	GG	0.62	0.87	0.79	0.75	0.78	0.62	0.87	0.74	0.83	0.74				
		(23)	(39)	(31)	(40)	(162)	(23)	(39)	(67)	(95)	(71)				

	GA	0.35	0.13	0.21	0.23	0.22	0.35	0.13	0.26	0.17	0.25	9.570	0.297	12.005	0.151
		(13)	(6)	(8)	(12)	(46)	(13)	(6)	(23)	(19)	(24)				
	AA	0.03	0.00	0.00	0.02	0.00	0.03	0.00	0.00	0.01	0.01				
		(1)	(0)	(0)	(1)	(1)	(1)	(0)	(0)	(1)	(1)				
SMAD5 (Oar_v4.0:Chr5:44907171:T>G)	TT	0.68	0.67	0.63	0.64	0.70	0.68	0.67	0.72	0.64	0.70				
		(25)	(30)	(25)	(34)	(147)	(25)	(30)	(65)	(74)	(67)				
	TG	0.32	0.27	0.35	0.30	0.27	0.32	0.27	0.28	0.31	0.27	5.910	0.657	8.496	0.387
		(12)	(12)	(14)	(16)	(57)	(12)	(12)	(25)	(36)	(26)				
	GG	0.00	0.07	0.03	0.06	0.02	0.00	0.07	0.00	0.05	0.03				
		(0)	(3)	(1)	(3)	(5)	(0)	(3)	(0)	(6)	(3)				
SMAD5 (Oar_v4.0:Chr5:44907730:G>A)	GG	0.36	0.40	0.38	0.38	0.27	0.36	0.40	0.26	0.36	0.27				
		(13)	(18)	(15)	(20)	(56)	(13)	(18)	(23)	(42)	(26)				
	GA	0.47	0.47	0.40	0.45	0.55	0.47	0.47	0.46	0.50	0.58	7.081	0.528	13.830	0.086
		(17)	(21)	(16)	(24)	(115)	(17)	(21)	(41)	(58)	(56)				
	AA	0.17	0.13	0.23	0.17	0.18	0.17	0.13	0.29	0.14	0.15				
		(6)	(6)	(9)	(9)	(38)	(6)	(6)	(26)	(16)	(14)				
SMAD5 (Oar_v4.0:Chr5:44908255:A>G)	AA	0.86	0.78	0.83	0.83	0.89	0.86	0.78	0.83	0.87	0.90				
		(32)	(35)	(33)	(44)	(185)	(32)	(35)	(75)	(101)	(86)				
	AG	0.14	0.20	0.15	0.15	0.10	0.14	0.20	0.14	0.11	0.09	5.057	0.751	4.872	0.771
		(5)	(9)	(6)	(8)	(21)	(5)	(9)	(13)	(13)	(9)				
	GG	0.00	0.02	0.03	0.02	0.01	0.00	0.02	0.02	0.02	0.01				
		(0)	(1)	(1)	(1)	(3)	(0)	(1)	(2)	(2)	(1)				
FGF7 (Oar_v4.0:Chr7:57740946:G>A)	AA	0.32	0.25	0.30	0.15	0.14	0.32	0.25	0.18	0.17	0.14				
		(12)	(11)	(12)	(8)	(29)	(12)	(11)	(16)	(20)	(13)				

	AG	0.43	0.50	0.48	0.55	0.43	0.43	0.50	0.37	0.48	0.51	20.226	0.010*	14.048	0.081
		(16)	(22)	(19)	(29)	(90)	(16)	(22)	(33)	(56)	(49)				
	GG	0.24	0.25	0.23	0.30	0.43	0.24	0.25	0.46	0.34	0.35				
		(9)	(11)	(9)	(16)	(90)	(9)	(11)	(41)	(40)	(34)				
FGF7 (Oar_v4.0:Chr7:57741376:T>G)	GG	0.32	0.24	0.31	0.15	0.14	0.32	0.24	0.18	0.17	0.14				
		(12)	(11)	(12)	(8)	(29)	(12)	(11)	(16)	(20)	(13)				
	GT	0.43	0.51	0.46	0.55	0.43	0.43	0.51	0.37	0.48	0.51	20.449	0.009*	14.105	0.079
		(16)	(23)	(18)	(29)	(90)	(16)	(23)	(33)	(55)	(49)				
	TT	0.24	0.24	0.23	0.30	0.43	0.24	0.24	0.46	0.35	0.35				
		(9)	(11)	(9)	(16)	(90)	(9)	(11)	(41)	(40)	(34)				
FGF7 (Oar_v4.0:Chr7:57803771:C>T)	CC	0.11	0.29	0.35	0.42	0.36	0.11	0.29	0.42	0.35	0.34				
		(4)	(13)	(14)	(22)	(75)	(4)	(13)	(38)	(40)	(33)				
	CT	0.38	0.27	0.10	0.25	0.35	0.38	0.27	0.24	0.28	0.36	25.468	0.001*	16.169	0.040*
		(14)	(12)	(4)	(13)	(72)	(14)	(12)	(22)	(32)	(35)				
	TT	0.51	0.44	0.55	0.34	0.29	0.51	0.44	0.33	0.37	0.29				
		(19)	(20)	(22)	(18)	(61)	(19)	(20)	(30)	(43)	(28)				
FGFR2 (Oar_v4.0:Chr22:40345986:C>T)	CC	0.76	0.69	0.65	0.81	0.74	0.76	0.69	0.79	0.72	0.73				
		(28)	(31)	(26)	(43)	(155)	(28)	(31)	(71)	(83)	(70)				
	CT	0.24	0.29	0.33	0.17	0.22	0.24	0.29	0.20	0.26	0.22	5.479	0.705	6.231	0.621
		(9)	(13)	(13)	(9)	(47)	(9)	(13)	(18)	(30)	(21)				
	TT	0.00	0.02	0.03	0.02	0.03	0.00	0.02	0.01	0.03	0.05				
		(0)	(1)	(1)	(1)	(7)	(0)	(1)	(1)	(3)	(5)				
FGFR2 (Oar_v4.0:Chr22:40346188:T>C)	TT	0.11	0.13	0.10	0.08	0.13	0.11	0.13	0.12	0.13	0.09				
		(4)	(6)	(4)	(4)	(27)	(4)	(6)	(11)	(15)	(9)				

	TC	0.41	0.49	0.46	0.53	0.45	0.41	0.49	0.51	0.44	0.46	2.764	0.948	3.020	0.933
		(15)	(22)	(18)	(28)	(95)	(15)	(22)	(46)	(51)	(44)				
	CC	0.49	0.38	0.44	0.40	0.42	0.49	0.38	0.37	0.43	0.45				
		(18)	(17)	(17)	(21)	(87)	(18)	(17)	(33)	(49)	(43)				
FGFR2 (Oar_v4.0:Chr22:40346469:G>C)	GG	0.35	0.25	0.38	0.34	0.28	0.35	0.25	0.31	0.31	0.28				
		(13)	(11)	(15)	(18)	(58)	(13)	(11)	(28)	(36)	(27)				
	GC	0.49	0.57	0.45	0.55	0.54	0.49	0.57	0.53	0.51	0.55	4.115	0.847	1.575	0.991
		(18)	(25)	(18)	(29)	(113)	(18)	(25)	(48)	(59)	(53)				
	CC	0.16	0.18	0.18	0.11	0.18	0.16	0.18	0.16	0.18	0.17				
		(6)	(8)	(7)	(6)	(38)	(6)	(8)	(14)	(21)	(16)				
FGFR2 (Oar_v4.0:Chr22:40346646:C>T)	CC	0.22	0.36	0.33	0.17	0.31	0.22	0.36	0.24	0.28	0.33				
		(8)	(16)	(13)	(9)	(64)	(8)	(16)	(22)	(32)	(32)				
	CT	0.51	0.49	0.48	0.64	0.50	0.51	0.49	0.57	0.53	0.47	7.762	0.457	5.098	0.747
		(19)	(22)	(19)	(34)	(104)	(19)	(22)	(51)	(61)	(45)				
	TT	0.27	0.16	0.20	0.19	0.20	0.27	0.16	0.19	0.20	0.20				
		(10)	(7)	(8)	(10)	(41)	(10)	(7)	(17)	(23)	(19)				
FGFR2 (Oar_v4.0:Chr22:40347084:G>A)	GG	0.32	0.16	0.21	0.25	0.24	0.32	0.16	0.22	0.23	0.25				
		(12)	(7)	(8)	(13)	(50)	(12)	(7)	(20)	(27)	(24)				
	GA	0.49	0.58	0.51	0.58	0.50	0.49	0.58	0.54	0.49	0.52	5.932	0.655	4.555	0.804
		(18)	(26)	(20)	(31)	(104)	(18)	(26)	(49)	(56)	(50)				
	AA	0.19	0.27	0.28	0.17	0.26	0.19	0.27	0.23	0.28	0.23				
		(7)	(12)	(11)	(9)	(55)	(7)	(12)	(21)	(32)	(22)				
FGFR2 (Oar_v4.0:Chr22:40348212:A>G)	AA	0.49	0.40	0.50	0.43	0.44	0.49	0.40	0.42	0.46	0.47				
		(18)	(18)	(20)	(23)	(93)	(18)	(18)	(38)	(53)	(45)				

	AG	0.43	0.51	0.40	0.51	0.46	0.43	0.51	0.49	0.46	0.45	2.196	0.974	1.107	0.997
		(16)	(23)	(16)	(27)	(97)	(16)	(23)	(44)	(53)	(43)				
	GG	0.08	0.09	0.10	0.06	0.09	0.08	0.09	0.09	0.09	0.08				
		(3)	(4)	(4)	(3)	(19)	(3)	(4)	(8)	(10)	(8)				
SMAD5 (Oar_v4.0:Chr5:44905960:T>G)	TT	0.22	0.44	0.28	0.34	0.26	0.22	0.44	0.18	0.34	0.19				
		(8)	(20)	(11)	(18)	(53)	(8)	(20)	(16)	(40)	(18)				
	TG	0.54	0.33	0.48	0.36	0.50	0.54	0.33	0.58	0.44	0.58	10.383	0.239	13.200	0.105
		(20)	(15)	(19)	(19)	(103)	(20)	(15)	(52)	(51)	(55)				
	GG	0.24	0.22	0.25	0.30	0.25	0.24	0.22	0.24	0.21	0.24				
		(9)	(10)	(10)	(16)	(51)	(9)	(10)	(22)	(24)	(23)				

Note: Values are presented as percentage (count). Percentages are calculated within each column (trait level), χ^2 values are Pearson Chi-Square statistics from SPSS, P values are Asymptotic Significance (2-sided), * indicates statistical significance at $P < 0.05$.
