# Supplementary material for: FGF7 Polymorphisms, Especially Chr7:57803771:C>T, are Associated with Lambskin Traits in Hu Sheep
Source: Animals (Basel). 2026 Oct 5;16(19):3126. doi: 10.3390/ani16193126 (PMC13663528; doi:10.3390/ani16193126)
Supplement: Supplementary file 1 [file animals-16-03126-s001.zip › Supplementary Table S5.pdf]

Supplementary Table S5. Associations between SNP genotypes and quantitative lambskin traits in Hu sheep

Gene	SNP	Trait	Genotype	N	Mean	SD	P
SOSTDC1	Oar_v4.0:Chr4:25022929:A>T	Pattern width	AA	207	1.482125604	0.644558495	0.41013646
SOSTDC1	Oar_v4.0:Chr4:25022929:A>T	Pattern width	AT	142	1.503521127	0.620959218	0.41013646
SOSTDC1	Oar_v4.0:Chr4:25022929:A>T	Pattern width	TT	34	1.338235294	0.81054217	0.41013646
SOSTDC1	Oar_v4.0:Chr4:25022929:A>T	Sacral wool length	AA	207	2.860386473	0.666228222	0.447573295
SOSTDC1	Oar_v4.0:Chr4:25022929:A>T	Sacral wool length	AT	142	2.906338028	0.642050658	0.447573295
SOSTDC1	Oar_v4.0:Chr4:25022929:A>T	Sacral wool length	TT	34	2.747058824	0.773514331	0.447573295
SMAD5	Oar_v4.0:Chr5:44852712:A>T	Pattern width	AA	190	1.532105263	0.635435766	0.270081868
SMAD5	Oar_v4.0:Chr5:44852712:A>T	Pattern width	AT	159	1.425157233	0.678603234	0.270081868
SMAD5	Oar_v4.0:Chr5:44852712:A>T	Pattern width	TT	33	1.418181818	0.618695475	0.270081868
SMAD5	Oar_v4.0:Chr5:44852712:A>T	Sacral wool length	AA	190	2.862631579	0.656931637	0.978539758
SMAD5	Oar_v4.0:Chr5:44852712:A>T	Sacral wool length	AT	159	2.870440252	0.669739554	0.978539758
SMAD5	Oar_v4.0:Chr5:44852712:A>T	Sacral wool length	TT	33	2.887878788	0.740674345	0.978539758
SMAD5	Oar_v4.0:Chr5:44852977:T>A	Pattern width	AT	202	1.421287129	0.663319203	0.17639327
SMAD5	Oar_v4.0:Chr5:44852977:T>A	Pattern width	AA	112	1.523214286	0.649460225	0.17639327
SMAD5	Oar_v4.0:Chr5:44852977:T>A	Pattern width	TT	68	1.572058824	0.619061781	0.17639327
SMAD5	Oar_v4.0:Chr5:44852977:T>A	Sacral wool length	AT	202	2.824752475	0.651734149	0.319458315
SMAD5	Oar_v4.0:Chr5:44852977:T>A	Sacral wool length	AA	112	2.941071429	0.680030944	0.319458315
SMAD5	Oar_v4.0:Chr5:44852977:T>A	Sacral wool length	TT	68	2.892647059	0.681814125	0.319458315
SMAD5	Oar_v4.0:Chr5:44905960:T>G	Pattern width	GG	110	1.57	0.621562104	0.063075123
SMAD5	Oar_v4.0:Chr5:44905960:T>G	Pattern width	TT	96	1.529166667	0.675108828	0.063075123
SMAD5	Oar_v4.0:Chr5:44905960:T>G	Pattern width	GT	176	1.396590909	0.652020835	0.063075123
SMAD5	Oar_v4.0:Chr5:44905960:T>G	Sacral wool length	GG	110	2.905454545	0.666613565	0.307005815
SMAD5	Oar_v4.0:Chr5:44905960:T>G	Sacral wool length	TT	96	2.929166667	0.685168695	0.307005815
SMAD5	Oar_v4.0:Chr5:44905960:T>G	Sacral wool length	GT	176	2.8125	0.658190159	0.307005815
SMAD5	Oar_v4.0:Chr5:44906216:C>T	Pattern width	CC	217	1.451152074	0.68408567	0.515338527

SMAD5	Oar_v4.0:Chr5:44906216:C>T	Pattern width	CT	142	1.497183099	0.60681048	0.515338527
SMAD5	Oar_v4.0:Chr5:44906216:C>T	Pattern width	TT	24	1.6	0.621358959	0.515338527
SMAD5	Oar_v4.0:Chr5:44906216:C>T	Sacral wool length	CC	217	2.846543779	0.643988972	0.593252154
SMAD5	Oar_v4.0:Chr5:44906216:C>T	Sacral wool length	CT	142	2.880985915	0.700246471	0.593252154
SMAD5	Oar_v4.0:Chr5:44906216:C>T	Sacral wool length	TT	24	2.9875	0.68608055	0.593252154
SMAD5	**Oar_v4.0:Chr5:44906657:G>A**	**Pattern width**	**GG**	**295**	**1.521016949**	**0.637409977**	**0.022872884**
SMAD5	**Oar_v4.0:Chr5:44906657:G>A**	**Pattern width**	**AG**	**85**	**1.349411765**	**0.679219357**	**0.022872884**
SMAD5	**Oar_v4.0:Chr5:44906657:G>A**	**Pattern width**	**AA**	**3**	**0.833333333**	**0.723417814**	**0.022872884**
SMAD5	Oar_v4.0:Chr5:44906657:G>A	Sacral wool length	GG	295	2.869830508	0.672444752	0.861727626
SMAD5	Oar_v4.0:Chr5:44906657:G>A	Sacral wool length	AG	85	2.855294118	0.657648387	0.861727626
SMAD5	Oar_v4.0:Chr5:44906657:G>A	Sacral wool length	AA	3	3.066666667	0.550757055	0.861727626
SMAD5	Oar_v4.0:Chr5:44907171:T>G	Pattern width	GT	111	1.436036036	0.70101132	0.526854044
SMAD5	Oar_v4.0:Chr5:44907171:T>G	Pattern width	TT	261	1.488505747	0.639607804	0.526854044
SMAD5	Oar_v4.0:Chr5:44907171:T>G	Pattern width	GG	12	1.641666667	0.372847357	0.526854044
SMAD5	Oar_v4.0:Chr5:44907171:T>G	Sacral wool length	GT	111	2.823423423	0.662358727	0.323784256
SMAD5	Oar_v4.0:Chr5:44907171:T>G	Sacral wool length	TT	261	2.896551724	0.675923575	0.323784256
SMAD5	Oar_v4.0:Chr5:44907171:T>G	Sacral wool length	GG	12	2.65	0.425334093	0.323784256
SMAD5	Oar_v4.0:Chr5:44907730:G>A	Pattern width	AA	122	1.518032787	0.694133194	0.688559577
SMAD5	Oar_v4.0:Chr5:44907730:G>A	Pattern width	GG	68	1.435294118	0.601432297	0.688559577
SMAD5	Oar_v4.0:Chr5:44907730:G>A	Pattern width	AG	193	1.475647668	0.635149031	0.688559577
SMAD5	**Oar_v4.0:Chr5:44907730:G>A**	**Sacral wool length**	**AA**	**122**	**2.875409836**	**0.668699425**	**0.015200664**
SMAD5	**Oar_v4.0:Chr5:44907730:G>A**	**Sacral wool length**	**GG**	**68**	**3.061764706**	**0.717797088**	**0.015200664**
SMAD5	**Oar_v4.0:Chr5:44907730:G>A**	**Sacral wool length**	**AG**	**193**	**2.79119171**	**0.634572633**	**0.015200664**
SMAD5	Oar_v4.0:Oar_v4.0:Chr5:44908255:A>G	Pattern width	AG	49	1.408163265	0.571488092	0.258688462
SMAD5	Oar_v4.0:Chr5:44908255:A>G	Pattern width	AA	329	1.481458967	0.664465904	0.258688462
SMAD5	Oar_v4.0:Chr5:44908255:A>G	Pattern width	GG	6	1.866666667	0.413118224	0.258688462
SMAD5	Oar_v4.0:Chr5:44908255:A>G	Sacral wool length	AG	49	2.975510204	0.619990125	0.413390395
SMAD5	Oar_v4.0:Chr5:44908255:A>G	Sacral wool length	AA	329	2.849240122	0.670598363	0.413390395

SMAD5	Oar_v4.0:Chr5:44908255:A>G	Sacral wool length	GG	6	3	0.81240384	0.413390395
FGF7	**Oar_v4.0:Chr7:57740946:G>A**	**Pattern width**	**AG**	**176**	**1.517613636**	**0.65576737**	**0.002097929**
FGF7	**Oar_v4.0:Chr7:57740946:G>A**	**Pattern width**	**GG**	**135**	**1.554814815**	**0.574289765**	**0.002097929**
FGF7	**Oar_v4.0:Chr7:57740946:G>A**	**Pattern width**	**AA**	**72**	**1.238888889**	**0.730146726**	**0.002097929**
FGF7	Oar_v4.0:Chr7:57740946:G>A	Sacral wool length	AG	176	2.797727273	0.676626468	0.170167233
FGF7	Oar_v4.0:Chr7:57740946:G>A	Sacral wool length	GG	135	2.931111111	0.642693338	0.170167233
FGF7	Oar_v4.0:Chr7:57740946:G>A	Sacral wool length	AA	72	2.916666667	0.6811341	0.170167233
FGF7	**Oar_v4.0:Chr7:57741376:T>G**	**Pattern width**	**GT**	**176**	**1.521909091**	**0.655680473**	**0.002154212**
FGF7	**Oar_v4.0:Chr7:57741376:T>G**	**Pattern width**	**TT**	**135**	**1.554814815**	**0.574289765**	**0.002154212**
FGF7	**Oar_v4.0:Chr7:57741376:T>G**	**Pattern width**	**GG**	**72**	**1.248888889**	**0.730146726**	**0.002154212**
FGF7	Oar_v4.0:Chr7:57741376:T>G	Sacral wool length	GT	176	2.8	0.67696803	0.181108077
FGF7	Oar_v4.0:Chr7:57741376:T>G	Sacral wool length	TT	135	2.931111111	0.642693338	0.181108077
FGF7	Oar_v4.0:Chr7:57741376:T>G	Sacral wool length	GG	72	2.916666667	0.6811341	0.181108077
FGF7	**Oar_v4.0:Chr7:57803771:C>T**	**Pattern width**	**CC**	**128**	**1.621875**	**0.498175806**	**0.003934208**
FGF7	**Oar_v4.0:Chr7:57803771:C>T**	**Pattern width**	**CT**	**115**	**1.464347826**	**0.675795996**	**0.003934208**
FGF7	**Oar_v4.0:Chr7:57803771:C>T**	**Pattern width**	**TT**	**140**	**1.358571429**	**0.730820542**	**0.003934208**
FGF7	Oar_v4.0:Chr7:57803771:C>T	Sacral wool length	CC	128	2.94453125	0.745570499	0.217864315
FGF7	Oar_v4.0:Chr7:57803771:C>T	Sacral wool length	CT	115	2.796521739	0.611000105	0.217864315
FGF7	Oar_v4.0:Chr7:57803771:C>T	Sacral wool length	TT	140	2.855714286	0.632031261	0.217864315
FGFR2	Oar_v4.0:Chr22:40345986:C>T	Pattern width	TT	283	1.458657244	0.654570104	0.391820129
FGFR2	Oar_v4.0:Chr22:40345986:C>T	Pattern width	CT	91	1.512087912	0.653679358	0.391820129
FGFR2	Oar_v4.0:Chr22:40345986:C>T	Pattern width	CC	10	1.72	0.518116246	0.391820129
FGFR2	Oar_v4.0:Chr22:40345986:C>T	Sacral wool length	TT	283	2.885512367	0.677894636	0.670113308
FGFR2	Oar_v4.0:Chr22:40345986:C>T	Sacral wool length	CT	91	2.821978022	0.625886429	0.670113308
FGFR2	Oar_v4.0:Chr22:40345986:C>T	Sacral wool length	CC	10	2.78	0.73303024	0.670113308
FGFR2	Oar_v4.0:Chr22:40346188:T>C	Pattern width	CT	178	1.517977528	0.646688531	0.517998637
FGFR2	Oar_v4.0:Chr22:40346188:T>C	Pattern width	TT	160	1.4375	0.670281083	0.517998637
FGFR2	Oar_v4.0:Chr22:40346188:T>C	Pattern width	CC	45	1.46	0.61103489	0.517998637

FGFR2	Oar_v4.0:Chr22:40346188:T>C	Sacral wool length	CT	178	2.888202247	0.700464836	0.410684815
FGFR2	Oar_v4.0:Chr22:40346188:T>C	Sacral wool length	TT	160	2.820625	0.645360498	0.410684815
FGFR2	Oar_v4.0:Chr22:40346188:T>C	Sacral wool length	CC	45	2.957777778	0.605838596	0.410684815
FGFR2	Oar_v4.0:Chr22:40346469:G>C	Pattern width	CG	203	1.502955665	0.64653772	0.509031522
FGFR2	Oar_v4.0:Chr22:40346469:G>C	Pattern width	CC	115	1.419130435	0.665819065	0.509031522
FGFR2	Oar_v4.0:Chr22:40346469:G>C	Pattern width	GG	65	1.506153846	0.648767322	0.509031522
FGFR2	Oar_v4.0:Chr22:40346469:G>C	Sacral wool length	CG	203	2.85270936	0.691053185	0.901232945
FGFR2	Oar_v4.0:Chr22:40346469:G>C	Sacral wool length	CC	115	2.88	0.646610596	0.901232945
FGFR2	Oar_v4.0:Chr22:40346469:G>C	Sacral wool length	GG	65	2.889230769	0.634459188	0.901232945
FGFR2	Oar_v4.0:Chr22:40346646:C>T	Pattern width	CT	198	1.477777778	0.648474282	0.593983025
FGFR2	Oar_v4.0:Chr22:40346646:C>T	Pattern width	TT	76	1.419736842	0.719262537	0.593983025
FGFR2	Oar_v4.0:Chr22:40346646:C>T	Pattern width	CC	110	1.519090909	0.608878568	0.593983025
FGFR2	Oar_v4.0:Chr22:40346646:C>T	Sacral wool length	CT	198	2.881313131	0.65802681	0.810158034
FGFR2	Oar_v4.0:Chr22:40346646:C>T	Sacral wool length	TT	76	2.823684211	0.673274272	0.810158034
FGFR2	Oar_v4.0:Chr22:40346646:C>T	Sacral wool length	CC	110	2.873636364	0.681024225	0.810158034
FGFR2	Oar_v4.0:Chr22:40347084:G>A	Pattern width	AG	199	1.496984925	0.647287233	0.265756343
FGFR2	Oar_v4.0:Chr22:40347084:G>A	Pattern width	AA	90	1.382222222	0.700012484	0.265756343
FGFR2	Oar_v4.0:Chr22:40347084:G>A	Pattern width	GG	94	1.527659574	0.611257167	0.265756343
FGFR2	Oar_v4.0:Chr22:40347084:G>A	Sacral wool length	AG	199	2.859798995	0.661717966	0.611455923
FGFR2	Oar_v4.0:Chr22:40347084:G>A	Sacral wool length	AA	90	2.828888889	0.683882578	0.611455923
FGFR2	Oar_v4.0:Chr22:40347084:G>A	Sacral wool length	GG	94	2.923404255	0.665982261	0.611455923
FGFR2	Oar_v4.0:Chr22:40348212:A>G	Pattern width	AG	179	1.507821229	0.654925376	0.69690175
FGFR2	Oar_v4.0:Chr22:40348212:A>G	Pattern width	AA	172	1.448837209	0.65431437	0.69690175
FGFR2	Oar_v4.0:Chr22:40348212:A>G	Pattern width	GG	33	1.46969697	0.628234057	0.69690175
FGFR2	Oar_v4.0:Chr22:40348212:A>G	Sacral wool length	AG	179	2.900558659	0.69042384	0.574571896
FGFR2	Oar_v4.0:Chr22:4034821:2A>G	Sacral wool length	AA	172	2.827906977	0.647740321	0.574571896
FGFR2	Oar_v4.0:Chr22:40348212:A>G	Sacral wool length	GG	33	2.896969697	0.636143089	0.574571896

N indicates the number of animals within each genotype group. Values are presented as mean ± SD. Different superscript lowercase letters within the same

SNP and trait indicate significant differences between genotype groups according to Tukey's HSD post hoc test ($P < 0.05$). *P* values represent the overall genotype effect from one-way analysis of variance.
